# Global analysis of iron metabolism‐related genes identifies potential mechanisms of gliomagenesis and reveals novel targets

**DOI:** 10.1111/cns.14386

**Published:** 2023-08-07

**Authors:** Jiayue Zhang, Liang Zhao, Shurui Xuan, Zhiyuan Liu, Zhenkun Weng, Yu Wang, Kexiang Dai, Aihua Gu, Peng Zhao

**Affiliations:** ^1^ Department of Neurosurgery The First Affiliated Hospital of Nanjing Medical University Nanjing China; ^2^ Department of Neurosurgery The Affiliated Brain Hospital of Nanjing Medical University Nanjing China; ^3^ Department of Respiratory and Critical Care Medicine The First Affiliated Hospital of Nanjing Medical University Nanjing China; ^4^ State Key Laboratory of Reproductive Medicine, School of Public Health Nanjing Medical University Nanjing China; ^5^ Key Laboratory of Modern Toxicology of Ministry of Education Center for Global Health, Nanjing Medical University Nanjing China

**Keywords:** Bioinformatic analysis, biomarkers, glioma, iron metabolism, therapeutic targets, TMZ resistance

## Abstract

**Aims:**

This study aimed to investigate key regulators of aberrant iron metabolism in gliomas, and evaluate their effect on biological functions and clinical translational relevance.

**Methods:**

We used transcriptomic data from multiple cross‐platform glioma cohorts to identify key iron metabolism‐related genes (IMRGs) based on a series of bioinformatic and machine learning methods. The associations between IMRGs and prognosis, mesenchymal phenotype, and genomic alterations were analyzed in silico. The performance of the IMRGs‐based signature in predicting temozolomide (TMZ) treatment sensitivity was evaluated. In vitro and in vivo experiments were used to explore the biological functions of these key IMRGs.

**Results:**

HMOX1, LTF, and STEAP3 were identified as the most essential IMRGs in gliomas. The expression levels of these genes were strongly related to clinicopathological and molecular features. The robust IMRG‐based gene signature could be used for prognosis prediction. These genes facilitate mesenchymal transformation, driver gene mutations, and oncogenic alterations in gliomas. The gene signature was also associated with TMZ resistance. HMOX1, LTF, and STEAP3 knockdown in glioma cells significantly reduced cell proliferation, colony formation, migration, and malignant invasion.

**Conclusion:**

The study presented a comprehensive view of key regulators underpinning iron metabolism in gliomas and provided new insights into novel therapeutic approaches.

## Graphical abstract

The mechanisms underlying aberrant iron metabolism in gliomas are not fully understood. The present study identified three key iron metabolism‐related genes (HMOX1, LTF, and STEAP3) based on a series of bioinformatic and machine learning methods. Comprehensive analysis using single‐cell RNAseq and multi‐omics data further indicated that the key regulators were significantly associated with mesenchymal phenotypes and TMZ sensitivity. In vitro and in vivo studies confirmed that the three genes contributed to gliomagenesis and chemotherapy resistance.

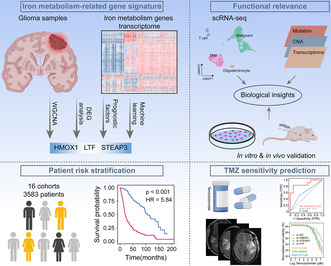

## 1 INTRODUCTION

Gliomas are common and aggressive neoplastic tumors of the central nervous system. According to the CBTRUS statistical report, gliomas accounted for approximately 25% of all adult primary brain tumors and 81% of malignant central nervous system tumors in the United States between 2013 and 2017.^1^ Based on their origin, gliomas can be classified into various histopathological types, including astrocytomas, oligodendrogliomas, oligoastrocytomas, and ependymomas. The 2016 WHO central nervous system tumor classification further stratified gliomas by incorporating genome‐based criteria, including IDH and ATRX mutations and 1p/19q codeletions.^2^ Despite advances in standard therapy, which includes surgical resection combined with temozolomide (TMZ)‐based chemotherapy and radiotherapy, tumors inevitably recur. Although not as intensively investigated to date, glioma relapse may be attributed to the infiltration of residual malignant cells into normal brain tissue and the progressive development of chemoresistance.^3^

Clinical studies have indicated that not all glioma patients benefit from TMZ treatment.^4^, ^5^ O6‐alkylguanine DNA alkyltransferase, encoded by O6 methylguanine‐DNA‐methyltransferase (MGMT), can reduce the effectiveness of TMZ therapy.^6^ Genome‐wide analyses have revolutionized our understanding of the high tumor heterogeneity and have revealed potential molecular subtypes of gliomas based on specific genetic alterations. The molecular classification of gliomas proposed by Verhaak et al., which includes proneural, neural, classical, and mesenchymal, is widely accepted.^7^ Selective effects of chemotherapy often result in subtype shifts over time, such as proneural phenotype transitions into the mesenchymal type, which was explained by the acquisition of TMZ resistance.^8^ Novel treatment approaches targeting oncogenic signaling pathways have also been developed. A phase II trial for glioblastoma (GBM) patients with MGMT unmethylation reported that the mTOR inhibitor temsirolimus could prolong overall survival in patients with mTOR^Ser2448^ phosphorylation.^9^ Bevacizumab, a monoclonal antibody targeting vascular endothelial growth factor signaling, has been shown to effectively improve GBM prognosis.^10^ The mitogen‐activated protein kinase inhibitor selumetinib showed meaningful antitumor activity in low‐grade glioma (LGG) patients with BRAF‐V600E mutations.^11^ Further efforts are required to develop promising therapeutic drugs that target the drivers and genomic aberrations of diffuse gliomas.

Iron is an essential element for various basic functions of human cells. Numerous enzymes involved in DNA replication, repair, and transport are dependent on cellular iron.^12^ Like a double‐edged sword, the excessive iron load is strongly associated with increased reactive oxygen species and thus prompts carcinogenesis.^13^ Behr et al. found that ^68^Ga‐citrate, a Fe^3+^ biomimetic that can bind to transferrin, enhanced PET/MR imaging of high‐grade gliomas and assisted tumor diagnosis.^14^ Zhou et al. found that the iron chelator, Dp44mT, inhibited glioma cell proliferation and promoted apoptosis through the IL6/JAK2/STAT3 signaling pathway.^15^ Masui et al. demonstrated that mTORC2 regulates iron metabolism in glioma cells by regulating histone H3 acetylation in the promoter regions of iron‐related genes, which is responsible for their malignant characteristics.^16^ However, the molecular mechanisms of iron metabolism in gliomas have not been extensively investigated and there is a lack of clinical trials targeting iron metabolism alone or in combination with the standard‐of‐care treatment.

The present study investigates the role of aberrant iron metabolism in gliomagenesis. Large‐scale gene expression profiles of glioma patients from multi‐center cohorts were used to identify three key iron metabolism‐related genes (IMRGs) through bioinformatic and machine learning approaches. Furthermore, we constructed an independent prognostic signature based on these three genes, which showed a satisfactory ability to predict the overall survival of glioma patients. We also demonstrated that the IMRG signature was strongly related to the mesenchymal phenotype. In addition, we examined the cellular biological functions of these key genes using a series of in vitro experiments. Together, our results provide a deeper insight into the regulators underpinning the abnormal iron metabolism in gliomas and the implications for potential therapeutic targets.

## 2 MATERIALS AND METHODS

### 2.1 RNA‐seq cohorts

The Cancer Genome Atlas (TCGA) pan‐glioma transcriptome profiling data with both raw counts and transcripts per kilobase million (TPM) values were obtained from the UCSC Xena data portal (https://xena.ucsc.edu/). This cohort was a combination of the TCGA‐LGG and TCGA‐GBM datasets. A total of five normal brain samples, 522 LGG, and 165 GBM tissues were included in the dataset. Additional expression profiles of 291 normal brain tissues from the Genotype‐Tissue Expression (GTEx) cohort were downloaded from the UCSC Toil project, which recomputed the RNAseq data from the TCGA and GTEx datasets to ensure the absence of batch effects.^17^ GTEx normal samples were selected on the basis of anatomic position using the UCSCXenaTools R package, including the cortex, frontal cortex (Ba9), and anterior cingulate cortex (Ba24).^18^ The corresponding curated survival data for glioma patients were obtained from published literature.^19^

The Chinese Glioma Genome Atlas (CGGA) RNA‐seq cohort included 182 LGG and 139 GBM samples. Gene expression data with fragments per kilobase million (FPKM) values were downloaded from the official portal (https://www.cgga.org.cn/) and converted to TPM values to allow comparison of gene expression levels between samples.

The Yanovich cohort (GSE149009) included 68 GBM samples with transcriptome and follow‐up data. Normalized TPM‐formatted gene expression data were derived from the original publication. Gene symbols in the expression data matrix were annotated using the biomaRt R package.^20^

### 2.2 Microarray cohorts

Gene expression and clinical data of glioma patients from 10 independent glioma cohorts, including REMBRANDT (GSE108474), Gravendeel (GSE16011), Kamoun (E‐MTAB‐3892), Lee (GSE13041), Phillips (GSE4271), Frejie (GSE4412), Gorovets (GSE35158), Joo (GSE42669), Ducray (E‐TABM‐898), and Weller (GSE61374), were obtained from the GeneExpression Omnibus (GEO) (https://www.ncbi.nlm.nih.gov/geo/) and ArrayExpress Microarray Database (https://www.ebi.ac.uk/arrayexpress/). All probe‐level data were normalized using the built‐in normalizeBetweenArrays function in the limma R package.^21^ Probe IDs of the gene expression data were reannotated to update the mapped gene symbols using R packages corresponding to the microarray platforms. Other datasets were annotated using the provided GPL files when matching R packages were unavailable.

### 2.3 NanoString RNA counting cohorts

The study also included the AVAglio (GSE150604), ATE (GSE149921), and GLARIUS (GSE150612) cohorts. These datasets consisted of 349, 452, and 123 GBM samples, respectively. Gene expression data were quantified using paraffin‐embedded tumor blocks based on NanoString nCounter platforms. The AVAglio and ATE assays were performed using a custom panel of 786 genes, while the GLARIUS dataset was based on a panel of 814 genes. Raw expression data were downloaded and normalized using the RUVSeq algorithm in the NanoNormIter R package.^22^

### 2.4 IMRGs

The definition of IMRGs was based on two previous studies.^23^, ^24^ Zhang et al. systematically selected 70 IMRGs based on the related Reactome (iron uptake and transport) and Gene Ontology (cellular iron ion homeostasis) gene terms, along with a literature review.^23^ Miller et al. identified 61 IMRGs from several Gene Ontology categories relevant to iron homeostasis and a manual literature search.^24^ A total of 88 genes were finally selected as IMRGs for the next analyses after combining these independent gene sets.

### 2.5 Weighted gene co‐expression network analysis (WGCNA)

The weighted co‐expression network was developed based on the gene expression data and TPM values of the TCGA pan‐glioma cohort using the WGCNA R package.^25^ Samples without complete data for survival status and overall survival time were excluded. Insufficiently abundant genes with TPM <1 in over 75% of the samples were removed from the expression matrix. Genes with the top 5000 median absolute deviation (MAD) values were filtered to avoid potential noise from nonvarying genes. Prior to the WGCNA algorithm, the glioma samples were clustered using hierarchical clustering based on the average linkage method, and obvious outliner samples were removed. A pairwise correlation matrix of selected genes was calculated and converted to a weighted adjacency matrix based on the optimal soft threshold. The topological overlap matrix (TOM) was then constructed to measure network connectivity, which represents the connection strength of each gene with other network genes. Finally, clusters of genes with high absolute correlations were further divided into distinct gene modules using the integrated dynamic tree‐cutting function.

To evaluate the stability of the identified gene modules in external glioma cohorts, module preservation analysis was performed 200 times with the permutation test procedure using the WGCNA R package. Two statistics, Z_summary_ and median rank, were computed for each glioma dataset. Z_summary_ was a summarized index of both density preservation and network connectivity, while relative preservation among the modules was compared using median rank.^26^ Generally, modules with Z_summary_ > 10 and relatively lower median rank indices indicated strong preservation.

### 2.6 Key gene selection and model construction

Preliminary screening was performed to identify differentially expressed IMRGs between glioma and normal brain samples using the DESeq2 R package, where IMRGs with |log2 fold change| >1 and *p*‐value <0.05 were considered statistically significant genes.^27^ Next, univariate Cox regression analysis was used to analyze the associations between differentially expressed IMRGs and the prognosis of glioma patients. Genes with *p*‐values <0.01 were selected as prognostic factors. Random survival forest analysis was performed to narrow down key IMRGs using the randomForestSRC R package. Random survival forest trees (*n* = 1000) were grown based on the log‐rank splitting rule, and the prediction error was used to measure the performance of the predictor for ranking survival in two random individuals. Finally, IMRGs with relatively high variable importance were selected for model development. A risk model was developed using multivariate Cox regression analysis, and the risk score for each sample was calculated as the sum of the gene expression value and the corresponding regression coefficient. The prognostic value of the risk model was evaluated using Kaplan–Meier survival analysis.

### 2.7 Molecular subtype prediction

Pre‐defined gene centroids for Verhaak's glioma classification were obtained from the original publication.^7^ Gene expression data for the test glioma datasets were normalized using z‐scores, and the subtype of each sample was predicted using the clusterRepro R package. The in‐group proportion statistics for the subtypes were calculated to reflect the clustering quality. Regarding IGS glioma classification, we determined the subtype‐specific genes by combining the differential expression gene analysis with the prediction analysis of microarray (PAM) algorithm.^28^ Gene profiling data from the original source of IGS classification (GSE16011) were downloaded and normalized. Silhouette width was computed to remove less representative samples based on the IGS class information, and only samples with positive silhouette widths were reserved. Differentially expressed genes (adjusted *p*‐value <0.05) among different IGS subtypes were calculated using the limma R package.^21^ The ability of differentially expressed genes to distinguish between subtypes was measured using the area under the curve (AUC); genes with an AUC >0.85 were used for building the classifier. Centroids were further determined by minimizing prediction errors <2% using 10‐fold cross‐validation for 1000 iterations. Finally, the IGS classification for the test datasets was estimated using the clusterRepro method.

### 2.8 Gene set enrichment analysis (GSEA)

GSEA analysis was performed to interpret the enrichment of signaling pathways and predefined gene terms at the gene level. Gene sets (H, C2, and C5 collections) were downloaded from the Molecular Signatures Database (v7.5.1, https://www.gsea‐msigdb.org/gsea). Two TMZ‐resistance‐related gene terms, “glioma therapy resistance” and “GBM TMZ nonresponse”, were obtained from previous studies.^29^, ^30^ The enrichment score for each gene term was calculated using the clusterProfiler R package with default parameters.^31^ Gene sets with adjusted *p*‐values <0.05 were considered significantly enriched.

### 2.9 Cancer cell line data

Transcriptome profiling data for GBM cells were downloaded from the GEO database (GSE23806 and GSE91392) and the human glioblastoma cell culture (HGCC) cohort. These three datasets were based on Affymetrix microarray platforms. We normalized the expression profiles of these cohorts and then classified these cells into neural, proneural, classical, and mesenchymal subtypes using the Verhaak glioma classification centroids. The AUCs for dose–response curves of 13 GBM cell lines from GSE91392 that received TMZ treatment were derived from the original publication.^32^ AUC index measures the efficacy of treatment; lower AUC values indicate greater sensitivity to the drug.

The protein‐coding gene expression data for 1406 human cancer cell lines were obtained from the Cancer Cell Line Encyclopedia (CCLE, https://depmap.org/). The CERES scores of the 1086 cancer cell lines with CRISPR‐Cas9 gene knockout were also downloaded. CERES indicates gene dependency levels in specific cancer cells; negative scores indicate that the corresponding genes are likely to have an essential role in neoplastic cell growth and survival. Drug sensitivity data for the cancer cell lines were obtained from the Profiling Relative Inhibition Simultaneously in Mixtures (PRISM, https://depmap.org/repurposing/) database and the Cancer Therapeutics Response Portal (CTRP, https://portals.broadinstitute.org/ctrp/). PRISM included the AUC values of 480 drugs used to treat 1492 cancer cell lines, while CTRP generated data from 835 cell lines using 481 small molecules and drugs.

### 2.10 Single‐cell RNA‐seq (scRNA‐seq) analysis

Raw scRNA‐seq data for a high‐grade glioma was downloaded from the GEO database (GSE185231). The Seurat (v4.0.6) standardized workflow was carried out. Eligible cells were screened using the following criteria: 500 < nFeature_RNA < 5500; 1000 < nCount_RNA < 25,000; and mitochondrial reads <20%. Then, the expression matrix was log normalized and the most variable 2000 genes were filtered using the FindVariableFeatures function. Principal component analysis was performed for dimension reduction, and the optimal clustering number was determined using the FindClusters function (with the top 10 principal components and resolution = 0.2). The identified clusters were visualized using UMAP reduction. The cells were annotated into different clusters using cluster‐overexpressed genes. InferCNV, a computational method for inferring chromosomal copy number alterations (CNA) from the scRNA‐seq data, was used to identify malignant cells at the recommended parameter settings.^33^ Oligodendrocytes were considered normal cells and used as a reference. The enrichment score for the gene set in each cell was quantified using the AUCell R package.^34^ The pseudotime trajectory analysis was performed to estimate developmental trajectories and the potential evolution process of malignant cells using the Monocle2 algorithm.^35^

### 2.11 Somatic mutation and CNA analysis

Somatic mutation data for LGG and GBM samples called by the MuTect2 pipeline were downloaded from the TCGA portal. The mutation landscapes in different risk groups were visualized using the oncoplot function in the maftools R package.^36^ Significantly mutated genes (*q* < 0.05) were obtained using the MutSigCV method. Co‐occurrence and exclusive mutations in the high‐ and low‐risk groups, respectively, were identified using the Rediscover package.^37^ Pre‐processed CNA data for the TCGA glioma cohort were obtained from Firehose (https://gdac.broadinstitute.org/). The relationship between the risk score and CNAs of the glioma samples was analyzed using GISTIC with default parameters (v2.0, https://www.genepattern.org/). GISTIC analysis was also performed in the high‐ and low‐risk groups to identify significant alteration peaks in each subpopulation (*q* < 0.25).

### 2.12 TMZ sensitivity estimation

The pRRophetic R package's built‐in ridge regression model was used to predict the AUC values for GBM samples in the TCGA cohort.^38^ Since the drug sensitivity data for both the PRISM and CTRP datasets were available, this computational method estimated the TMZ response in GBM patients based on the gene expression profiles. Before proceeding, contaminated glioma cell lines and those with missing AUC values were excluded from further analysis. Genes with low variability (MAD <0.4) were filtered out from both the training expression data and the TCGA GBM expression matrix. The AUC values of TMZ treatment for each GBM sample were estimated using the pRRophetic package with default parameters.

### 2.13 MRI data collection and processing

The Ivy Glioblastoma Atlas Project (Ivy‐GAP) included genomic data, detailed clinical information, and MRI images of GBM patients. Gene expression and clinical data were downloaded from the Ivy‐GAP site (https://ivygap.org/), while restricted MRI data were obtained from The Cancer Imaging Archive (TCIA).^39^ We excluded patients who consistently received less than three cycles of TMZ treatment after tumor resection. Patients with multifocal GBM with incomplete resections were also removed from the dataset. Patients with both contrast‐enhanced T1‐weighted and T2‐weighted FLAIR images performed close to the dates of post‐surgery and the last TMZ cycle administration (or before the start of another chemotherapeutic regimen) were included. We only took into account the period when the patients consistently received TMZ treatment alone if other drugs were administered concurrently or between TMZ cycles. Imaging sequences in DICOM format that met the above‐mentioned criteria were extracted and converted into TIFF files using the Python script. The images were reviewed by two senior radiologists. The selected patients were stratified into TMZ responders and non‐responders based on progression‐free survival (PFS; cutoff: 6 months) and radiological assessment.

### 2.14 In vitro and in vivo experimental procedures

Sample collection, immunohistochemistry (IHC) staining, cell culture and treatment, quantitative real‐time polymerase chain reaction, siRNA transfection, immunofluorescence staining, cell viability assays, colony formation assays, flow cytometry, wound closure assays, transwell invasion assays, Western blotting, and an in vivo xenograft model are detailed in Data S1.

### 2.15 Statistical analyses

All statistical analyses were performed using the R software (v4.2.0, The R foundation www.r‐project.org). The pooled hazard ratio (HR) of the risk model in glioma cohorts was computed using meta‐analysis. Statistical heterogeneity was evaluated using Cochran's Q test and I‐squared statistics. The random‐effects model was used if heterogeneity was significant with a *p*‐value of Q < 0.05 and I‐squared >50%. Otherwise, a fixed‐effect model was applied. Based on the multivariate Cox proportional hazards regression model, a nomogram was developed to quantify the survival likelihood of each patient by incorporating the risk model and clinicopathological features. The tumor purity of the glioma sample was inferred using the ESTIMATE algorithm based on gene expression data.^40^ Other tumor purity indices (CPE, LUMP, and InfiniumPurify) and intratumor heterogeneity (ITH) scores (DEPTH, DITHER, sITH, and gITH), which were calculated from different omics data of the TCGA glioma cohort, were obtained from the original publications.^41^, ^42^, ^43^, ^44^, ^45^ Subclass mapping (SubMap), an unsupervised method to assess the correspondence of gene expression profiles between independent datasets, was performed using the GenePattern module with default parameters.^46^

To assess the normality of continuous variables, the Shapiro–Wilk test was employed. For data that followed a normal distribution, the Student's *t*‐test was employed to compare the differences between two groups. In cases the data was not normally distributed, the Wilcoxon rank‐sum test was used. The Kruskal–Wallis test was applied for comparisons among more than two groups. Pearson's correlation was used to assess correlations between continuous variables. Both univariate and multivariate Cox regression models were constructed using the survival R package. Fisher's exact test was used to compare the proportions of TMZ respondents among the groups. A two‐tailed *p*‐value <0.05 was considered statistically significant for all hypothetical tests.

## 3 RESULTS

### 3.1 Identification of key IMRGs in gliomas

**FIGURE 1:**
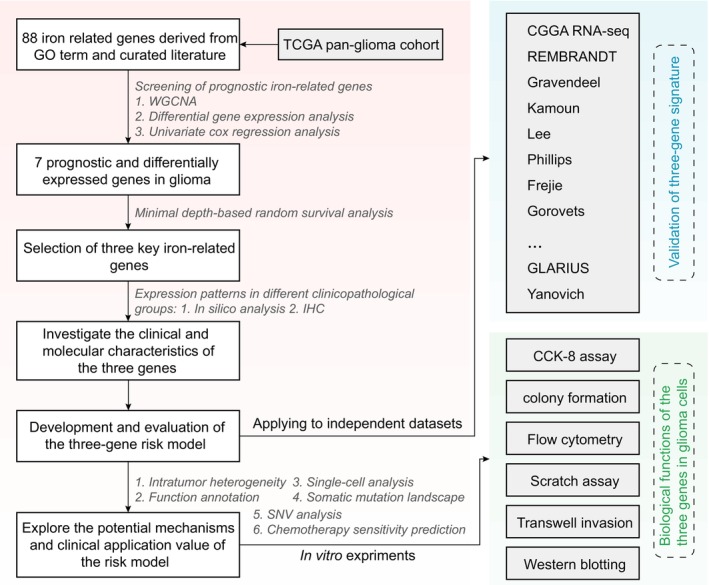
Workflow of the study. Iron metabolism‐related genes (IMRGs) were obtained by manual literature searches. TCGA pan‐glioma cohort was used to identify both prognostic and differentially expressed key IMRGs. Molecular features of the filtered genes were explored, and a risk model was developed. Fifteen independent external glioma datasets were included to validate the prognostic value of the gene signature. Both in silico analyses and in vitro experiments confirmed that the gene signature was strongly associated with tumor heterogeneity, mesenchymal phenotype, TMZ resistance, and progression of gliomagenesis.

The flow chart of the study design is shown in Figure 1. Detailed information on all 16 glioma cohorts is listed in Table S1. The transcriptome data for three cohorts were based on high‐throughput sequencing; ten cohorts were based on microarrays, and the remaining three cohorts were profiled using the NanoString nCounter platform. The clinical and pathological characteristics of the 3892 glioma patients are listed in Table S2. To reduce the potential statistical bias due to the insufficient normal sample size in the TCGA glioma RNA‐seq dataset (*n* = 5), the TCGA GTEx combined dataset from the UCSC Toil RNA‐seq Recompute project, which contains a large amount of normal human brain gene expression data, was used.

**FIGURE 2:**
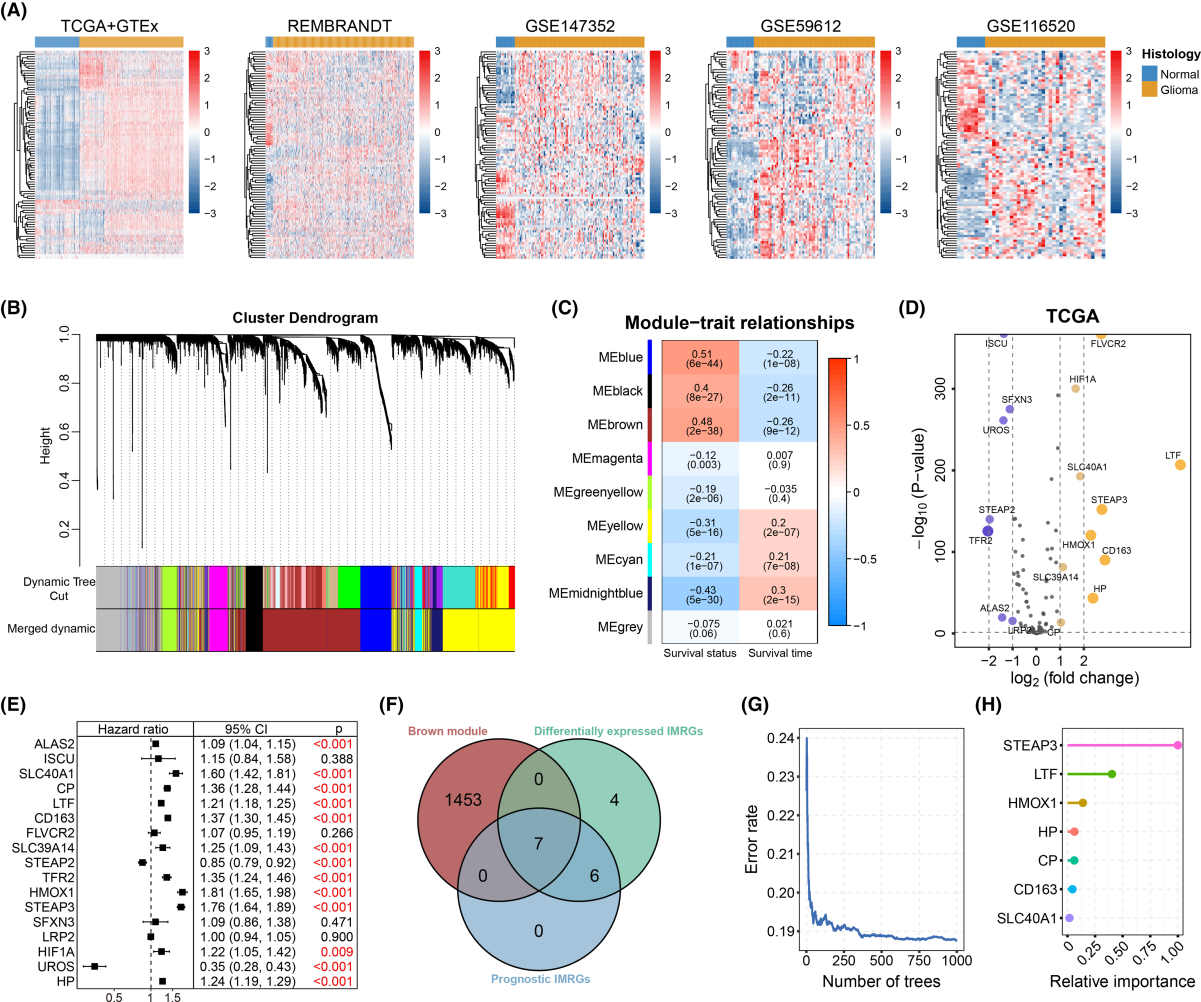
IMRG expression patterns in gliomas and normal brain tissues and identification of key genes. (A) Heatmaps of IMRG expression values (Z‐scored by row) for glioma and normal brain samples in TCGA+GTEx, REMBRANDT, GSE147352, GSE59612, and GSE116520 datasets. Genes were clustered based on Spearman correlation. Upper bar: glioma, yellow; normal brain, blue. (B) Clustering dendrogram for genes built using WGCNA analysis. Colored bars below the tree represent gene modules with highly similar gene expression profiles; merged bars are mixtures of gene modules highly similar to other modules. (C) Heatmaps for correlations between gene modules and survival status as well as survival time in glioma patients. The upper value in each cell represents the correlation coefficient, while the lower value is the *p*‐value. (D) Volcano plot of differentially expressed IMRGs in glioma samples compared to normal brain tissues in the TCGA cohort (|log2FC| > 1 and adjusted *p* < 0.05). (E) Forest plot for the univariate Cox regression analysis based on differentially expressed IMRGs in the TCGA cohort. (F) Venn plot of overlapping genes among the brown gene module, differentially expressed IMRGs, and prognostic IMRGs. (G) The estimated error rate for the random survival forest with the tree number set to 1000 in the TCGA glioma cohort. (H) The sorted relative importance of each gene calculated using the random survival forest algorithm.

The 88 IMRGs are listed in Table S3. The expression levels of these genes in the TCGA GTEx cohort were examined (Figure 2A). The distinct expression patterns of these genes between normal and glioma samples were detected. Four independent external datasets composed of both normal and glioma samples, including REMBRANDT, GSE147352, GSE59612, and GSE116520, were included to validate the expression differences (Figure 2A). Similar results in these datasets confirmed that the IMRGs were dysregulated in gliomas.

WGCNA analysis was performed to filter the gene modules strongly associated with glioma prognosis. A coexpression network was constructed using the TCGA dataset. Two outliner samples were identified and excluded after hierarchical clustering (Figure S1A). Samples with incomplete survival information were also filtered out, yielding 650 glioma samples. The soft threshold metric (βvalue) was set to 10 to ensure a scale‐free network (Figure S1B,C). Gene modules with similarities >0.7 were merged into a single module, and finally nine modules were identified (Figures 2B, S1D). The interactions between different gene modules are shown in Figure S1E. The correlation between clinical traits and module eigengenes were analyzed. Considering both survival status and survival time, the brown module was chosen as the key gene module for this study (Figure 2C). The stability and reproducibility of gene modules were further validated in external glioma cohorts using module preservation analysis (Figure S2A‐O). The brown module proved to be robust and reproducible in most of the validation datasets with Z_summary_ scores >10 and relatively lower median rank indices.

Since differential expression of vital genes was common in tumor samples, we performed differential expression gene analysis on IMRGs in the TCGA glioma cohort (Table S4). As shown in Figure 2D, 17 IMRGs were chosen as potential key genes modulating glioma development. The prognostic values of these IMRGs were further confirmed using univariate Cox proportional hazards regression analyses, and 13 genes with *p* < 0.01 were identified as prognostic factors (Figure 2E). Combining the results of the WGCNA gene module, seven genes were considered preliminary key IMRGs (Figure 2F). We then used a random survival forest algorithm with 1000 bootstraps to rank the importance of prognostic genes. Finally, the top three genes (STEAP3, LTF, and HMOX1) with the greatest importance were chosen as key prognostic IMRGs for gliomas (Figure 2G–H, Table S5).

### 3.2 Key IMRG expression patterns in gliomas

**FIGURE 3:**
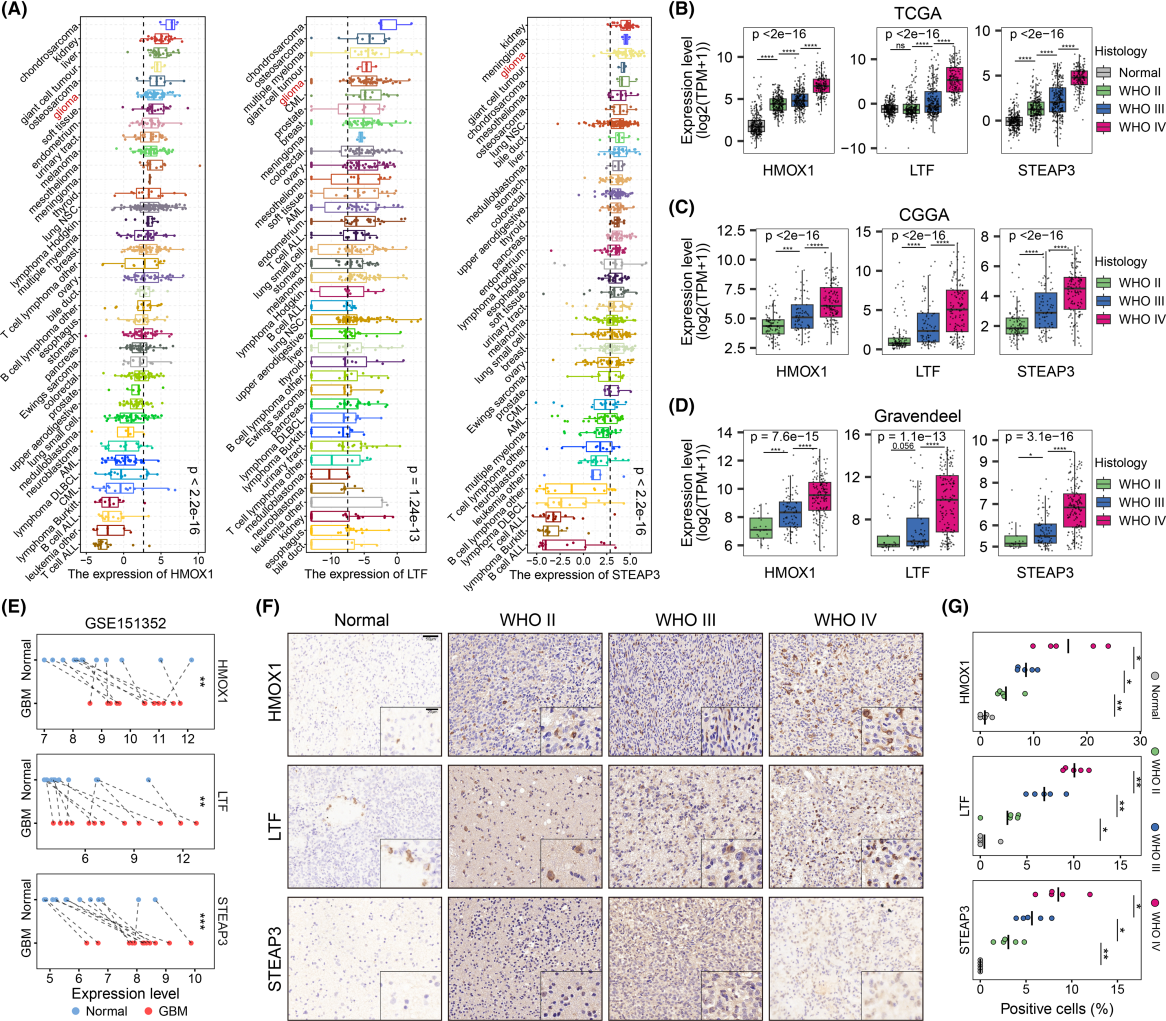
Expression levels of the three key IMRGs increased with increasing histological grade. (A) Variations in HMOX1, LTF, and STEAP3 expressions among 38 human cancer cell lines in the CCLE database. Overall *p*‐values were obtained using the Kruskal–Wallis test. HMOX1, LTF, and STEAP3 expression levels increased with the histological grade in TCGA (B), CGGA (C), and Gravendeel (D) cohorts. *P*‐values for pairwise comparisons were obtained using the Wilcoxon rank‐sum test; overall *p*‐values were obtained using the Kruskal–Wallis test. (E) Parallel axis dot plots showing expression differences of HMOX1, LTF, and STEAP3 between GBM and paired nontumor samples in the GSE151352 dataset. *P*‐values were calculated using the paired‐sample Wilcoxon rank‐sum test. (F) Representative images for IHC stained sections of HMOX1, LTF, and STEAP3 in glioma and normal brain specimens. Inset represents positive staining of target proteins. Scale bars: 50 μm and 20 μm (inset). (G) The proportions of IHC‐stained cells positive for HMOX1, LTF, and STEAP3. *P*‐values were calculated using the Student's *t*‐test. Black center lines represent the means for each group. *p < 0.05, **p < 0.1, ***p < 0.001, ****p < 0.0001.

The expression levels of the three IMRGs were compared among different human cancer cell lines using transcriptome data from the CCLE database. The data indicated that HMOX1, LTF, and STEAP3 had relatively higher expressions in glioma cells compared to most other cancer cell lines (Figure 3A). Similar expression landscapes were found as the results were validated in the GSE57083 dataset except for LTF (Figure S3A–C). We then investigated the association between the expression levels of these genes and clinicopathological features in the TCGA cohort. Glioma samples in general expressed the three genes at higher levels than normal brain tissues (Figure 3B). Moreover, the expression levels of all three IMRGs increased with increasing WHO tumor grade (Figure 3B). Likewise, the expression levels of most of the genes were strongly related to the tumor grade in the CGGA and Gravendeel cohorts, except for the LTF expression between grades Ι and ΙΙ in the Gravendeel dataset (*p* = 0.056) (Figure 3C–D). In the GSE15352 dataset, these three genes had higher expressions in GBM samples compared to the paired normal tissues (Figure 3E).

We also checked the protein levels of the three genes using proteomic data in the CPTAC dataset. The protein levels of these genes were significantly higher in GBM (*n* = 99) than in normal (*n* = 10) samples (Figure S3D). IHC staining of the three genes in normal brain tissues and grade II–IV gliomas indicated that the proteins were significantly upregulated in gliomas compared to normal samples (Figure 3F–G). Protein levels were also positively correlated with tumor grade in glioma samples.

### 3.3 Key IMRGs correlated with molecular features and anatomical heterogeneity in gliomas

**FIGURE 4:**
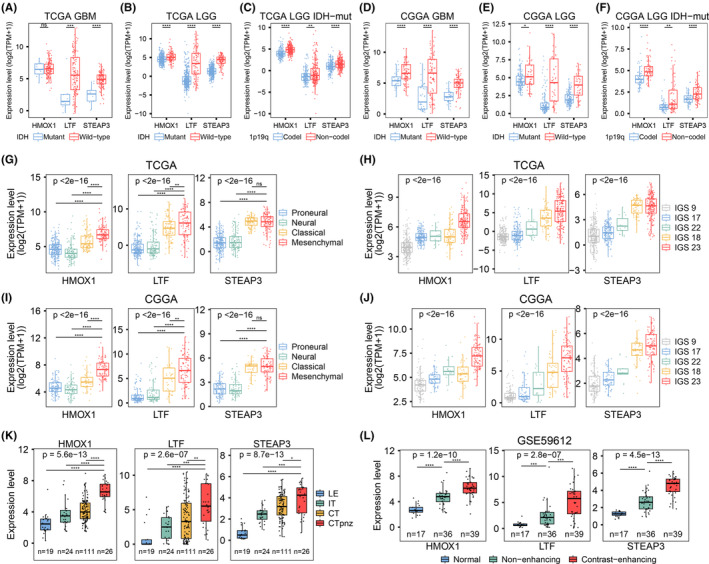
Expression patterns of key IMRGs among gliomas with distinct molecular features and cellular compositions. Boxplots for key IMRG expressions stratified by IDH mutation status in the TCGA GBM (A) and LGG (B) datasets. (C) Differences in mRNA levels of HMOX1, LTF, and STEAP3 between samples with 1p/19q codeletions and nondeletions in the TCGA IDH‐mutated LGG dataset. Boxplots for the three gene expressions stratified by IDH mutation status in the CGGA GBM (D) and LGG (E) datasets. (F) Differences in mRNA levels of HMOX1, LTF, and STEAP3 between samples with and without 1p/19q codeletions in the CGGA IDH‐mutated LGG dataset. Distribution of HMOX1, LTF, and STEAP3 expressions within different Verhaak (G) and IGS (H) subtypes in the TCGA and CGGA (I‐J) cohorts. (K) The boxplot shows HMOX1, LTF, and STEAP3 expressions in the four anatomic structures obtained from GBM samples in the Ivy‐GAP dataset. (L) Comparison of HMOX1, LTF, and STEAP3 expressions across distinct radiological regions of GBM samples. All *p*‐values for pairwise comparisons were calculated using the Wilcoxon rank‐sum test, while overall *p*‐values were calculated using the Kruskal–Wallis test. *p < 0.05, **p < 0.1, ***p < 0.001, ****p < 0.0001.

We also investigated whether the expression patterns of the three genes differed among gliomas with distinct molecular characteristics. In the TCGA GBM cohort, the expression levels of LTF and STEAP3 in IDH wild‐type samples (*n* = 147) were significantly higher than those with the IDH mutations (*n* = 11), while no statistical difference in HMOX1 expression existed between the groups (Figure 4A). In the TCGA LGG dataset, samples with IDH wild‐type phenotype (*n* = 96) expressed the three genes at higher levels than mutated samples (*n* = 423) (Figure 4B). Moreover, the expression levels of these three genes in IDH‐mutated LGG patients with 1p1q codeletions (*n* = 351) were higher than in those without combined deletions (*n* = 171) (Figure 4C). We also validated the findings in the CGGA cohort and found that the three genes were overexpressed in IDH wild‐type and 1p/19q codeletion glioma samples (Figure 4D–F).

In addition to the mutation characteristics and copy number variations, the associations between the three genes and molecular subtypes were further evaluated. The centroid of each glioma classification was calculated, and the glioma samples were stratified into Verhaak and IGS subtypes (Table S6). Based on the Verhaak classification, the mesenchymal subtype represented a worse prognosis and poor therapy response.^7^, ^47^ IGS 18, 22, and 23 subtypes predominantly resembled GBMs, while most of the gliomas in IGS 9 and 17 subtypes were LGGs.^48^ Here, mesenchymal glioma samples expressed higher levels of the three genes than those clustered into proneural, neural, and classical subtypes in the TCGA dataset (Figure 4G). The expression levels varied significantly across different IGS subtypes, with the highest level in IGS 23 and the lowest expression in IGS 9 (Figure 4H). As expected, similar results were found in the CGGA glioma cohort (Figure 4I–J).

The disparity in genomic features also existed among distinct anatomic structures and cellular compositions in GBM.^39^, ^49^ Using the transcriptome data from the Ivy‐GAP dataset, we found that the three genes were overexpressed in cellular tumor tissues of GBM, particularly in perinecrotic tumor zones, compared to leading edges and infiltrating tumors (Figure 4K). Contrast‐enhancing regions of GBM also had significantly higher levels of HOMX1, LTF, and STEAP3 compared to nonenhancing tissues with abnormal FLAIR signals (Figure 4L). Collectively, these three key IMRGs were closely related to the molecular phenotype of malignant glioma progression.

### 3.4 Construction and evaluation of a robust prognostic model based on transcriptome data

**FIGURE 5:**
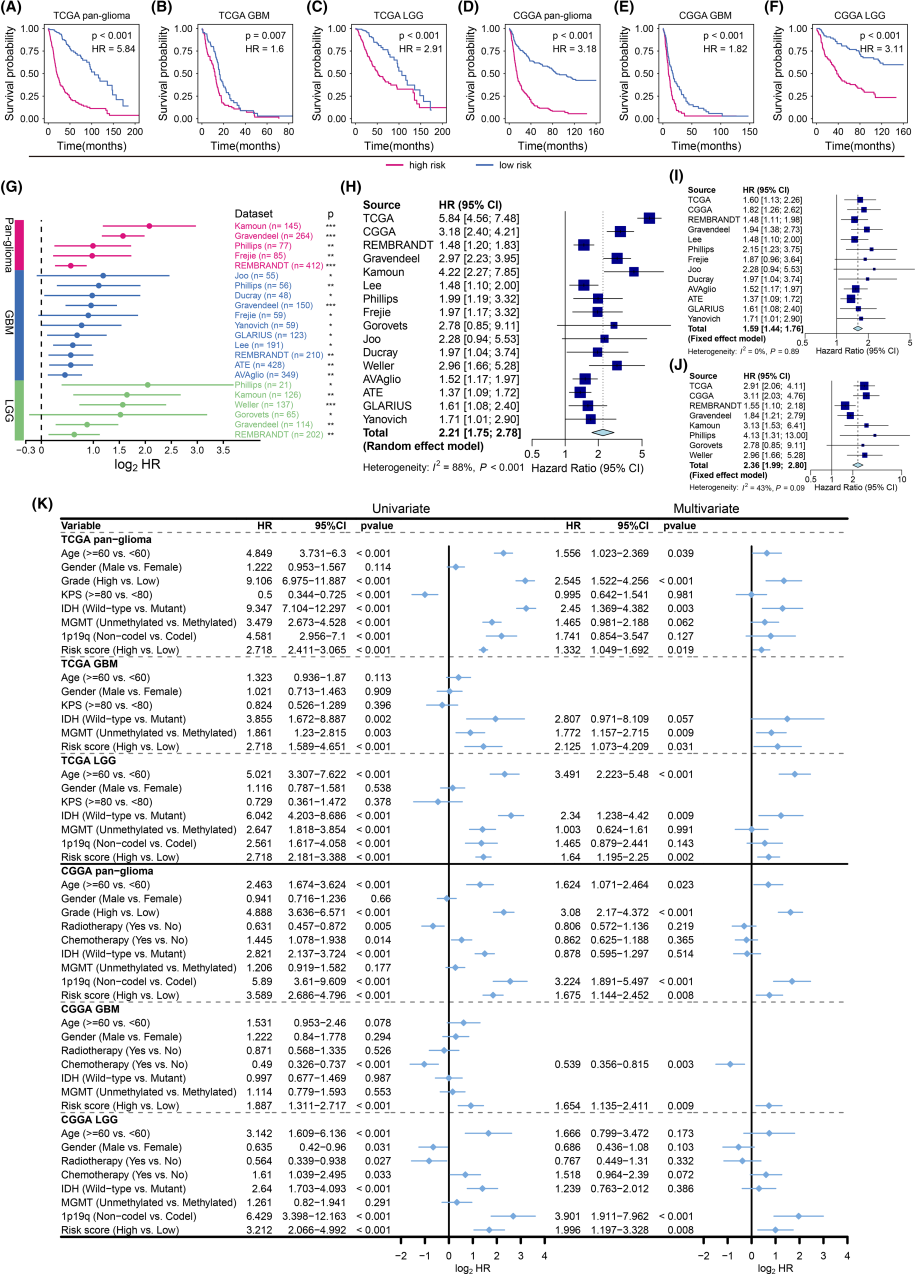
Development of a prognostic risk model based on the three key IMRGs in gliomas. Kaplan–Meier survival curves of patients with different risk scores in pan‐glioma, GBM, and LGG cohorts of the TCGA (A–C) and CGGA (D–F) datasets. (G) HRs and corresponding *p*‐values for the risk signature validated in external pan‐glioma, GBM, and LGG datasets (log‐rank test). The horizontal line range represents the 95% CI. *p < 0.05, **p < 0.1, ***p < 0.001. A forest plot showing the pooled HR values across independent pan‐glioma (H), GBM (I), and LGG (J) cohorts. (K) Univariate and multivariate analyses of clinicopathological characteristics and key IMRG‐based signature with overall survival in pan‐glioma, GBM, and LGG cohorts.

A total of 683 glioma samples in the TCGA cohort with complete overall survival information and RNA‐seq data were used to construct an IMRGs‐based risk model. The multivariate Cox regression analysis was performed to calculate the regression coefficient of each gene; each sample was assigned a risk score based on the combination of regression coefficients and the corresponding expression values of the three genes. Kaplan–Meier survival analysis indicated that patients with high risk scores had significantly worse prognoses compared to the low‐risk group in the pan‐glioma dataset (HR = 5.84, 95% confidence interval [CI]: 4.56–7.48, *p* < 0.001) (Figure 5A). Likewise, high‐risk groups showed significant survival disadvantages compared to low‐risk groups in both TCGA GBM (HR = 1.60, 95% CI: 1.13–2.26, *p* = 0.007) and LGG (HR = 2.91, 95% CI: 2.06–4.11, *p* < 0.001) subcohorts (Figure 5B,C). We further confirmed that the gene signature based on key IMRGs also effectively stratified pan‐glioma, GBM, and LGG patients into different risk groups with distinct prognoses in the CGGA cohort (Figure 5D–F). In an attempt to verify the generalizability and robustness of the risk model, 2584 glioma samples from 14 independent external datasets covering pan‐glioma, GBM, and LGG cohorts were used. The gene signature was consistently identified as an unfavorable molecular prognostic indicator across all cohorts (*p* < 0.05) (Figure 5G).

A meta‐analysis was performed to explore the comprehensive prognostic value of the gene signature in gliomas. Because of significant heterogeneity in the pan‐glioma cohort (*I* ^2^ = 88%, *p* < 0.001), a random‐effects model was used to estimate the pooled HR. The result showed that the risk signature is significantly associated with poor prognosis in glioma (combined HR = 2.21, 95% CI: 1.75–2.78, *p* < 0.0001) (Figure 5H). Both Egger's regression (*p* = 0.6721) and Begg's rank (*p* = 0.1497) tests were not statistically significant, which indicated no publication bias. Sensitivity analysis revealed that the pooled HR for the gene signature was not altered significantly after omitting a single study from the whole glioma cohort (Figure S4A). Since the HR value of the risk model varied for different glioma grades, we also performed meta‐analyses of the GBM and LGG subcohorts. The fixed‐effect models showed that the combined HRs of the risk gene signature were 1.59 (95% CI: 1.44–1.76, *p* < 0.0001) and 2.36 (95% CI: 1.99–2.80, *p* < 0.0001) in the GBM and LGG cohorts, respectively (Figure 5I–J). Sensitivity analysis using the leave‐one‐out method also confirmed the robust prognostic value of the risk model (Figure S4B,C).

The univariate and multivariate Cox regression analyses suggested that, along with clinical and molecular pathological features, the risk signature could function as an independent prognostic factor for pan‐glioma, GBM, and LGG datasets in both TCGA and CGGA cohorts (Figure 5K). Gravendeel, AVAglio, and Weller cohorts containing the essential molecular characteristics, such as IDH mutation, MGMT methylation, and 1p/19q codeletion status, were also included to validate the findings. High‐risk scores indicated a worse prognosis of gliomas independently (Table S7).

To identify high‐risk individuals and predict the prognosis of glioma patients quantitatively, we developed a nomogram based on the multivariable Cox proportional hazards model. The nomogram presented satisfactory C‐indices in both TCGA (0.8716, 95% CI: 0.846–0.897) and CGGA (0.7535, 95% CI: 0.722–0.785) glioma cohorts (Figure S5A–B). Calibration curves based on 1, 2, 3, and 5 year time points verified the agreement between the overall survival probability predicted by the nomogram and the actual observed outcomes in the TCGA and CGGA cohorts (Figure S5C–D).

### 3.5 IMRG‐based signature linked with tumor heterogeneity and mesenchymal phenotype

**FIGURE 6:**
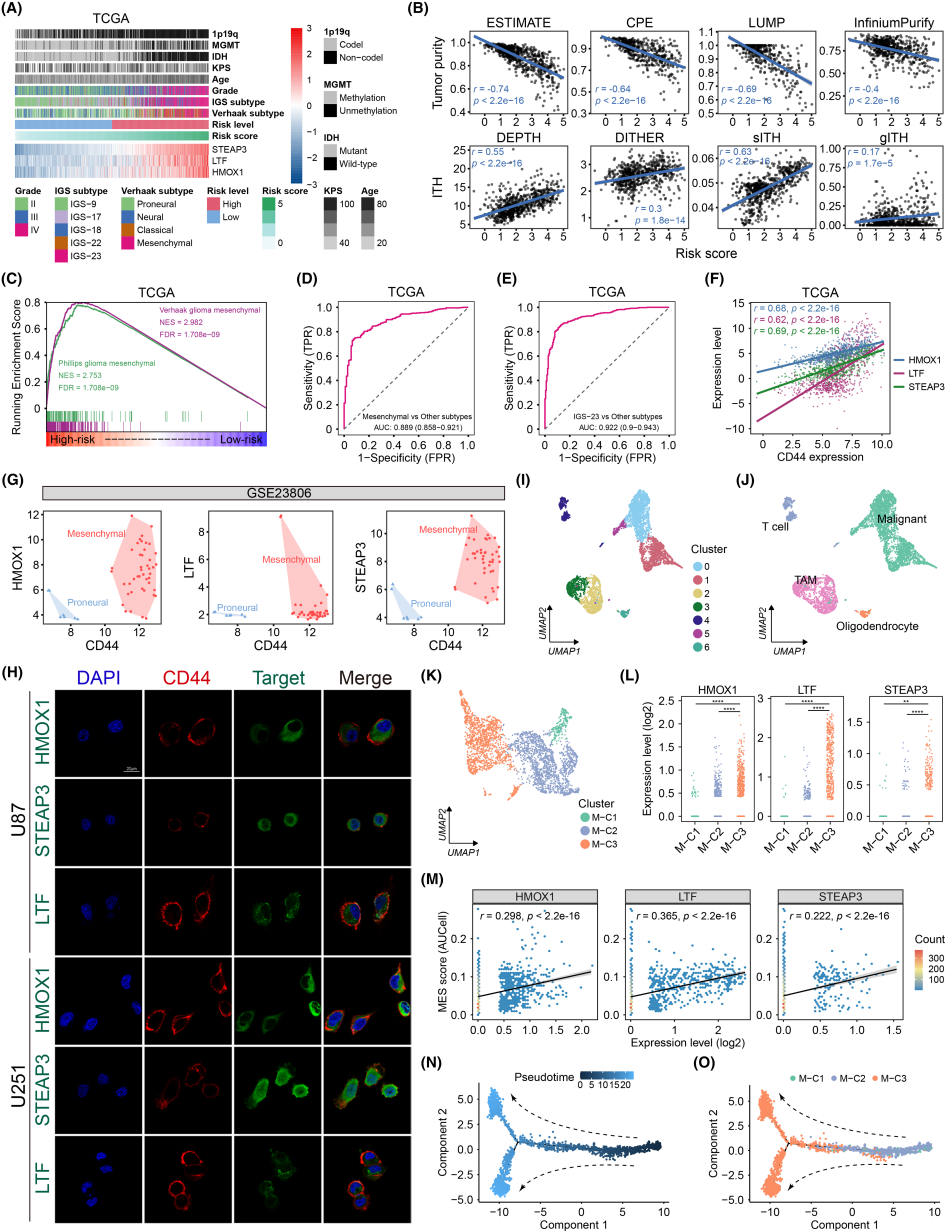
The gene signature was strongly associated with ITH and the mesenchymal phenotype. (A) An overview of the relationship of risk scores with clinicopathological and molecular characteristics in the TCGA glioma cohort. (B) Pearson correlation of risk scores with tumor purity (upper) and ITH index (lower) estimated using different algorithms. (C) GSEA enrichment plot of the “Verhaak glioma mesenchymal” and “Phillips glioma mesenchymal” gene terms. ROC curves measuring the performance of the gene signature for predicting mesenchymal (D) and IGS‐23 (E) subtypes. *X*‐axis: false‐positive rate (FPR); *y*‐axis: true‐positive rate (TPR). (F) Pearson correlation between the expressions of CD44 and the three signature genes. (G) Scatter plots showing the pair‐wise expression distribution of CD44 with HMOX1, LTF, and STEAP3 in the GSE23806 dataset. Red points represent mesenchymal subtype samples, while blue points represent proneural glioma cells. (H) Immunofluorescence confocal microscopy of CD44 (red) and the signature genes (green) in U87 and U251 glioma cells. Scale bar: 20 μm. (I) Reduced‐dimensionality (UMAP) visualization of single cells (*n* = 5529) in the GSE185231 dataset, colored by identified clusters. (J) UMAP plot of four annotated cell types in the transcriptomic space, colored by cell types. (K) UMAP visualization of three subpopulations of 3389 malignant glioma cells, colored by identified clusters. (L) HMOX1, LTF, and STEAP3 gene expressions in M‐C1 (*n* = 235), M‐C2 (*n* = 1664), and M‐C3 (*n* = 1490) clusters from the single‐cell dataset. *P*‐values were calculated using the Wilcoxon rank‐sum test. (M) Scatter plots of Pearson's correlations between the mesenchymal score and expression levels of the three signature genes in all malignant glioma cells. (N) Pseudotime trajectory of all malignant glioma cells analyzed using Monocle2. Each point corresponds to a single cell, and arrows represent inferred developmental directions. (O) Progression trajectory representation of malignant glioma cell populations, colored by clusters.

The significant difference in overall survival between high‐ and low‐risk glioma patients implied a strong association between the gene signature and tumor heterogeneity. The relationships between increasing risk scores and clinico‐molecular features in TCGA and CGGA cohorts were illustrated (Figure 6A, Figure S6A). In the TCGA glioma dataset, the high‐risk group had higher proportions of older, high‐grade, low KPS, IDH wild‐type, MGMT unmethylated, 1p/19q noncodel, mesenchymal, and IGS‐23 subtype patients (all *p* < 0.05) (Table S8). In addition, all unfavorable prognostic factors except for MGMT unmethylation (*p* = 0.069) were enriched in high‐risk patients in the CGGA cohort (Table S8). We obtained tumor purity estimates for all glioma samples in the TCGA dataset based on gene expression or methylation data using ESTIMATE, CPE, LUMP, and InfiniumPurify. The risk scores showed a consistently negative correlation with tumor purity derived from different algorithms (all *p* < 0.05) (Figure 6B). Additionally, the ITH index estimated using the DEPTH, DITHER, sITH, and gITH methods increased with increasing risk scores (all *p* < 0.05) (Figure 6B). Since the corresponding complete methylation and somatic mutation profiles of the CGGA glioma samples used in the study were unavailable, we calculated tumor purity using the ESTIMATE method. The risk score also correlated negatively with tumor purity in the CGGA cohort (*r* = −0.78, *p* < 0.0001) (Figure S6B).

GSEA analysis suggested that Verhaak glioma mesenchymal (NES = 2.982, FDR = 1.708e‐9) and Phillips glioma mesenchymal (NES = 2.753, FDR = 1.708e‐9) gene terms were significantly enriched in the TCGA high‐risk group (Figure 6C). In contrast, proneural glioma gene terms were enriched in low‐risk samples (Figure S6C). The results were further confirmed using the CGGA cohort (Figure S6D–E). Receiver operating characteristic (ROC) curves showed that the gene signature could satisfactorily distinguish between mesenchymal gliomas and other Verhaak subtypes in both TCGA (AUC = 0.889, 95% CI: 0.858–0.921) and CGGA (AUC = 0.923, 95% CI: 0.895–0.952) datasets (Figure 6D, Figure S6F). The AUC values were 0.922 (95% CI: 0.9–0.943) and 0.939 (95% CI: 0.914–0.964) for the identification of the IGS‐23 subtype in the TCGA and CGGA cohorts, respectively (Figure 6E, Figure S6G).

We also explored whether the three genes in the signature could function as effective independent biomarkers for mesenchymal gliomas. Novel phenotypic markers are commonly detected by checking whether the target gene is coexpressed with known phenotypic markers.^50^ The expression of CD44, a well‐characterized mesenchymal marker, was strongly positively correlated with HMOX1, LTF, and STEAP3 in both TCGA and CGGA datasets (all *p* < 0.0001) (Figure 6F, Figure S6H). Moreover, CD44 and the three genes were preferentially expressed in the mesenchymal group compared to the proneural subtype at the cellular level in the GSE23806, HGCC, and GSE91392 datasets (Figure 6G, Figure S6I–J). Consequently, immunofluorescence assays confirmed that the three genes were coexpressed with CD44 in U87 and U251 glioma cells (Figure 6H).

We next incorporated a high‐grade glioma scRNA‐seq dataset (GSE185231) to further reveal the association between these three genes and the mesenchymal glioma phenotype. After removing cells that failed the quality control process, a total of 5529 cells were selected. UMAP dimensionality reduction was used to identify seven independent cell clusters (Figure 6I). Cell type annotation combined with inferCNV analysis determined four cell types among these clusters, including malignant cells (n = 3389), oligodendrocytes (*n* = 169), T cells (*n* = 441), and tumor‐associated macrophages (*n* = 1530) (Figure 6J, Figure S7A–B). Neoplastic glioma cells were further divided into three clusters, M‐C1 (6.93%), M‐C2 (49.10%), and M‐C3 (43.97%), based on the UMAP dimensionality reduction analysis (Figure 6K). M‐C3 glioma cells were identified as mesenchymal‐like cells after matching differentially‐expressed genes with predefined gene meta‐modules of various malignant glioma cellular states.^51^ HMOX1, LTF, and STEAP3 were significantly overexpressed in the M‐C3 subtype at the single cell level (Figure 6L). The mesenchymal score, an index for the enrichment score of the Verhaak mesenchymal glioma gene term, was assigned to each malignant cell using the AUCell algorithm. We found that the expression levels of all three genes were positively correlated with mesenchymal scores (all *p* < 0.0001) (Figure 6M). The pseudotime trajectory analysis showed that pseudo‐temporal ordering began with the cells of the M‐C1 and M‐C2 groups and terminated with M‐C3 cells, indicating the potential transition direction of malignant glioma cells (Figure 6N–O). Based on the close association between the three genes and the mesenchymal phenotype, we concluded that HMOX1, LTF, and STEAP3 might drive mesenchymal transition and regulate the plasticity of malignant gliomas. In summary, HMOX1, LTF, and STEAP3 could act as potential biomarkers for the mesenchymal subtype.

### 3.6 Association of IMRG‐based signature with distinct mutational and CNA characteristics

**FIGURE 7:**
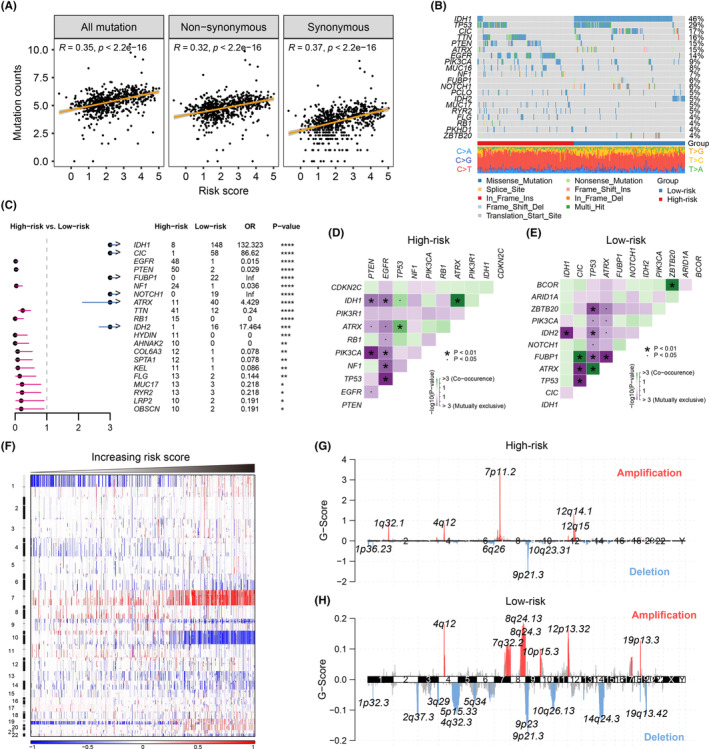
Distinct mutation patterns and copy number alterations between different risk‐level groups. (A) Pearson correlations of risk scores with total, non‐synonymous, and synonymous mutation counts. (B) Oncoplot shows the mutation landscape for the 20 most frequently mutated genes in the whole glioma samples. The annotation bar indicates the risk level, while the bottom panel represents the fraction of diverse mutation conversions within the samples. (C) Forest plot of differentially mutated genes between high‐risk and low‐risk samples. The horizontal line range represents the 95% CI of the odds ratio (OR). *P*‐values were calculated using Fisher's exact test. *p < 0.05, **p < 0.1, ***p < 0.001, ****p < 0.0001. Heatmaps of co‐occurrence and mutually exclusive mutations of the significantly mutated genes in the high‐ (D) and low‐risk (E) groups. (F) Association between relative copy numbers and an increasing risk score. The vertical annotation bar shows chromosome positions. (gains: red; losses: blue). Significantly amplified and deleted genomic peaks were identified using the GISTIC method in high‐risk (G) and low‐risk (H) groups.

We further investigated the genomic differences between high‐ and low‐risk groups after assessing transcriptional alterations. The risk score was positively correlated with all mutation (*r* = 0.35, *p* < 0.0001), non‐synonymous mutation (*r* = 0.32, *p* < 0.0001), and synonymous mutation (*r* = 0.37, *p* < 0.0001) counts in the TCGA cohort (Figure 7A). A total of 30 and 15 genes were mutated in more than 5% of the high‐ and low‐risk patients, respectively, with only six overlapping genes, including IDH1, TP53, ATRX, TTN, and MUC16 (Figure S8A,B). An oncoplot was built to show the 20 most frequently mutated genes in the whole cohort using the available mutation data (Figure 7B). The differentially mutated genes between the two risk groups were detected using the Fisher's exact test and illustrated as a forest plot (Figure 7C). For instance, mutations of IDH1, CIC, FUBP1, and NOTCH1 occurred preferentially in the low‐risk group, while EGFR, PTEN, and NF1 mutations were more common in the high‐risk group (all *p* < 0.05). Significantly mutated genes estimated using the MutSigCV module were defined as driver genes across different risk groups (all *q* < 0.05) (Table S9). Using Rediscover algorithm, more mutually exclusive mutations of these driver genes were identified than co‐occurrence mutations in the high‐ and low‐risk groups (Figure 7D–E).

Next, we investigated the CNA profiles between the two risk level groups in the TCGA dataset. The risk scores were positively correlated with total CNA numbers in glioma samples (*r* = 0.31, *p* < 0.0001) (Figure S8C). Notably, the gain of chr7 and loss of chr10 occurred simultaneously with increasing risk scores (Figure 7F). Furthermore, more samples carrying chr13/14 deletions and chr19/20 amplifications were found in patients with higher risk scores (Figure 7F). Significantly amplified and deleted gene regions in both high‐ and low‐risk groups were identified using the GISTIC module. A total of 11 regions of the genome were amplified and 26 regions deleted in the high‐risk group, while nine amplifications and 23 deletions were found in the low‐risk group (*q* < 0.25) (Table S10). In the samples with high risk scores, the representative genomic amplifications in cytobands included 7p11.2 (EGFR), 12q14.1 (CDK4), 4q12 (PDGFRA), 1q32.1 (PIK3C2B), and 12q15 (CPM, MDM2), while deletions included 9p21.3 (CDKN2A), 10q23.31 (PTEN), 1p32.3 (CDKN2C), and 6q26 (QKI) (Figure 7G). As for the low‐risk group, 8q24.13 (ZHX2), 4q12 (PDGFRA), and 12p13.32 (CCND2) were amplified, while 9p21.3 (CDKN2A), 14q24.3 (ACTN1), and 4q32.3 (NEK1) were deleted (Figure 7H). Although both 9p21.3 (CDKN2A) amplification and deletion were found in these two groups, the amplitudes and frequencies of the aberrations (G scores) in the low‐risk group were much lower than those in the high‐risk group.

### 3.7 The gene signature effectively predicted TMZ treatment response

**FIGURE 8:**
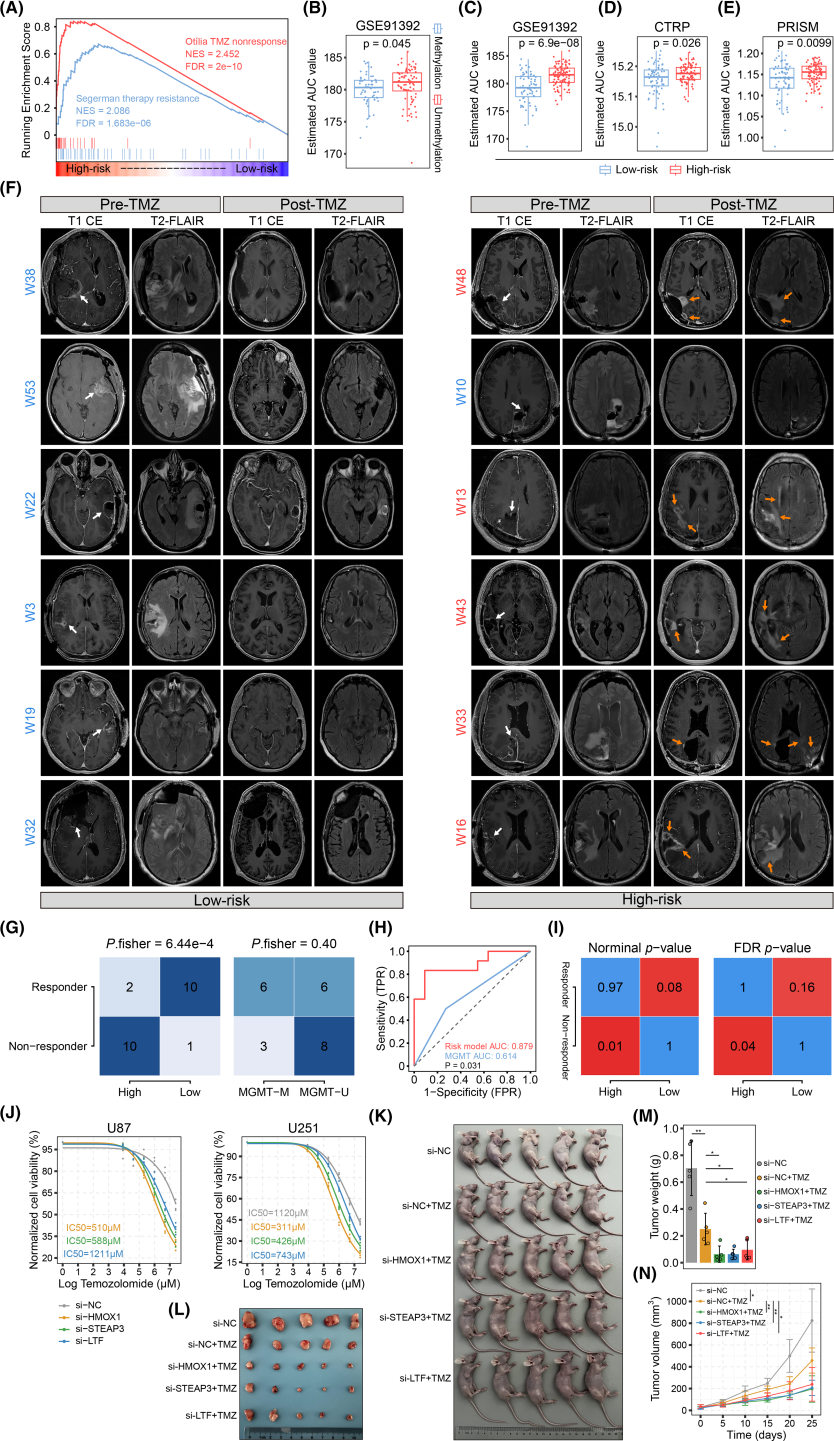
Association of the gene signature with TMZ resistance. (A) GSEA results of TMZ therapy resistance pathways in the TCGA GBM cohort. (B) The estimated AUC values in TCGA GBM patients with and without MGMT methylation. *P*‐values were calculated using the Wilcoxon rank‐sum test. Boxplots of estimated AUC values comparing high‐ and low‐risk GBM patients in the TCGA dataset based on drug sensitivity data from GSE91392 (C), CTRP (D), and PRISM (E). *P*‐values were calculated using the Wilcoxon rank‐sum test. (F) Representative axial contrast‐enhanced T1‐weighted (T1 CE) and T2‐weighted FLAIR MRI images of high‐risk (right panel) and low‐risk (left panel) GBM patients in the Ivy‐GAP cohort: pre‐ and post‐therapy (left to right). White arrows indicate the original enhanced tumor area before TMZ treatment, while orange arrows represent radiological features related to tumor progression. Blue font: responders; red font: non‐responders. (G) Confusion matrices comparing proportions of responders and non‐responders in different patient groups stratified by risk scores and MGMT methylation status. *P*‐values were calculated using Fisher's exact test. (H) ROC curves comparing the performance of the risk model and MGMT methylation in predicting TMZ therapy response. (I) SubMap analysis showing the consistency of transcriptome features between patients with different TMZ therapy responses in the Ivy‐GAP cohort and patients with different risk levels in the TCGA GBM dataset, colored by *p*‐values. (J) Cell viability of U87 and U251 glioma cells with and without knockdown of the three key IMRGs after treatment with TMZ at various concentrations. The dose‐response curves were fitted, and the IC50 values were calculated using the four‐parameter logistic nonlinear regression model. (K) Images of subcutaneous glioma xenograft‐bearing nude mice at day 25. (L) Tumor images and (M) tumor weights of mice from each group at day 25 (*n* = 5). *P*‐values were calculated using the Student's *t*‐test. (N) The tumor growth kinetics of tumor‐bearing mice in each group over the treatment process (*n* = 5). *P*‐values were calculated using the Student's *t*‐test. Data are presented as means ± standard deviation. *p < 0.05, **p < 0.1.

Pioneering investigations have reported that the proneural–mesenchymal transition allows the development of chemotherapeutic resistance in GBM by phenotypic plasticity, especially for TMZ.^30^ As previously demonstrated, the three‐gene signature was strongly associated with the mesenchymal phenotype. Therefore, we further explored whether the gene signature was responsible for chemoresistance in GBM. GSEA analysis showed that TMZ nonresponse (NES = 2.452, FDR = 2e‐10) and therapy resistance (NES = 2.086, FDR = 1.683e‐6) gene terms were significantly enriched in the TCGA high‐risk GBM group (Figure 8A). Next, we used the pRRophetic R package with an integrated ridge regression model to estimate the potential TMZ response in GBM patients based on the transcriptome profiles. Jiang et al. measured the dose‐response data for selected anti‐cancer agents, including TMZ, across human GBM cell lines,^32^ wherein the gene expression matrix and corresponding metadata were downloaded from the GSE91392 dataset. Before moving on, we assessed whether the TMZ response prediction procedure was feasible. MGMT methylation status is widely recognized as a classical molecular indicator to guide TMZ‐based chemotherapy in GBM patients. Therefore, GBM samples in the TCGA cohort were divided into two groups based on the MGMT methylation status (methylated: 56; unmethylated: 81). Based on the GSE91392 dataset, TCGA GBM patients with MGMT unmethylation were predicted to have higher AUC values compared to the methylated patients (*p* = 0.045), which was consistent with the clinical behavior of TMZ (Figure 8B). Meanwhile, the estimated AUC values in high‐risk patients were significantly higher than those in low‐risk patients (*p* = 6.9e‐8) (Figure 8C, Table S11).

The CTRP and PRISM datasets contain drug sensitivity data and gene expression profiles for human cancer cell lines and were used to further explore the relationship between TMZ sensitivity and the three‐gene signature. Uncontaminated glioma cells were obtained from the CTRP and PRISM datasets, which yielded 40 and 30 different cell lines, respectively. Cell lines that lacked the corresponding gene expression data were removed. Finally, 39 and 29 glioma cell lines were chosen from the CTRP and PRISM datasets, respectively. In agreement with the results from the GSE91392 dataset, high‐risk patients in the TCGA GBM cohort were predicted to have significantly higher AUC values using both the CTRP (*p* = 0.026) and PRISM (*p* = 0.0099) datasets (Figure 8D‐E, Table S11). This suggests that high‐risk GBM patients might benefit less from TMZ‐based treatment compared to low‐risk patients.

The Ivy‐GAPdataset containing transcriptome and MRI data, was used to further investigate whether the three‐gene signature could be clinically used as a biomarker for TMZ resistance. Based on the predefined selection criteria, 23 GBM patients were selected for the study. Since RNA‐sequenced tumor tissues contain different anatomic structures, only cellular tumor samples in the Ivy‐GAP dataset were used to eliminate the impact of nontumor components. GBM patients were stratified into different risk‐level groups based on the expression of the three genes in cellular tumor tissues. Based on the Response Assessment Neuro‐Oncology Group criteria, the imaging characteristics of both contrast‐enhanced T1‐weighted and T2‐weighted FLAIR sequences were compared before and after TMZ treatment. It is worth noting that all patients were TMZ responders in the first quartile low‐risk group (*n* = 6), with no obvious signs of tumor progression on MRI images (left panel of Figure 8F). However, five out of six patients in the fourth quartile high‐risk group developed significant GBM progression, as evidenced by increased enhancing or nonenhancing lesions (right panel of Figure 8F). Among all patients, there was a significant association between the risk model and TMZ response, with 10/11 responders and 10/12 nonresponders in the low‐ and high‐risk groups, respectively (*p* = 6.44e‐4) (Figure 8G). In contrast, there was no significant relationship between MGMT methylation status and TMZ response (*p* = 0.4) (Figure 8G). ROC analysis was performed to assess the sensitivity and specificity of the risk model signature and MGMT methylation status for predicting TMZ treatment response. The risk model signature was satisfactorily predictive of therapy response with an AUC of 0.879, whereas MGMT methylation was less predictive with an AUC of 0.614 (*p* = 0.031) (Figure 8H). In addition, we used the SubMap, an unsupervised algorithm for estimating the association significance between subclasses, to predict TMZ response in TCGA high‐ and low‐risk GBM patients. High‐risk patients were more likely to develop chemoresistance to TMZ treatment (*p* = 0.01, FDR = 0.04) (Figure 8I).

We also validated the impact of these three genes on TMZ sensitivity through in vitro experiments. While the control group did not achieve 50% inhibition under the maximum concentration of TMZ treatment, the half‐maximal inhibitory concentration (IC50) values of TMZ decreased significantly after knockdown of HMOX1 (IC50 = 510 μM), STEAP3 (IC50 = 588 μM), and LTF (IC50 = 1211 μM) in U87 glioma cells (Figure 8J). Similarly, compared to the control group (IC50 = 1120 μM), inhibiting the expression of these three genes increased the sensitivity of the U251 cells to TMZ (si‐HMOX1: IC50 = 311 μM, si‐STEAP3: IC50 = 426 μM, si‐HMOX1: IC50 = 743 μM). Considering that U87 cells exhibited greater resistance to TMZ compared to U251 cells, we chose U87 cells for the construction of the xenograft model in this study. The results showed that the knockdown of key genes (HMOX1, STEAP3, and LTF) combined with TMZ treatment significantly decreased both tumor volume and tumor weight compared to the group treated with TMZ alone (Figure 8K–N), indicating an effective restoration of sensitivity to TMZ therapy.

Altogether, these findings indicated that the three‐gene signature was significantly related to TMZ resistance in GBM. Moreover, this signature was a powerful molecular indicator for predicting the clinical efficacy of TMZ.

### 3.8 Cellular biological functions of the signature genes

**FIGURE 9:**
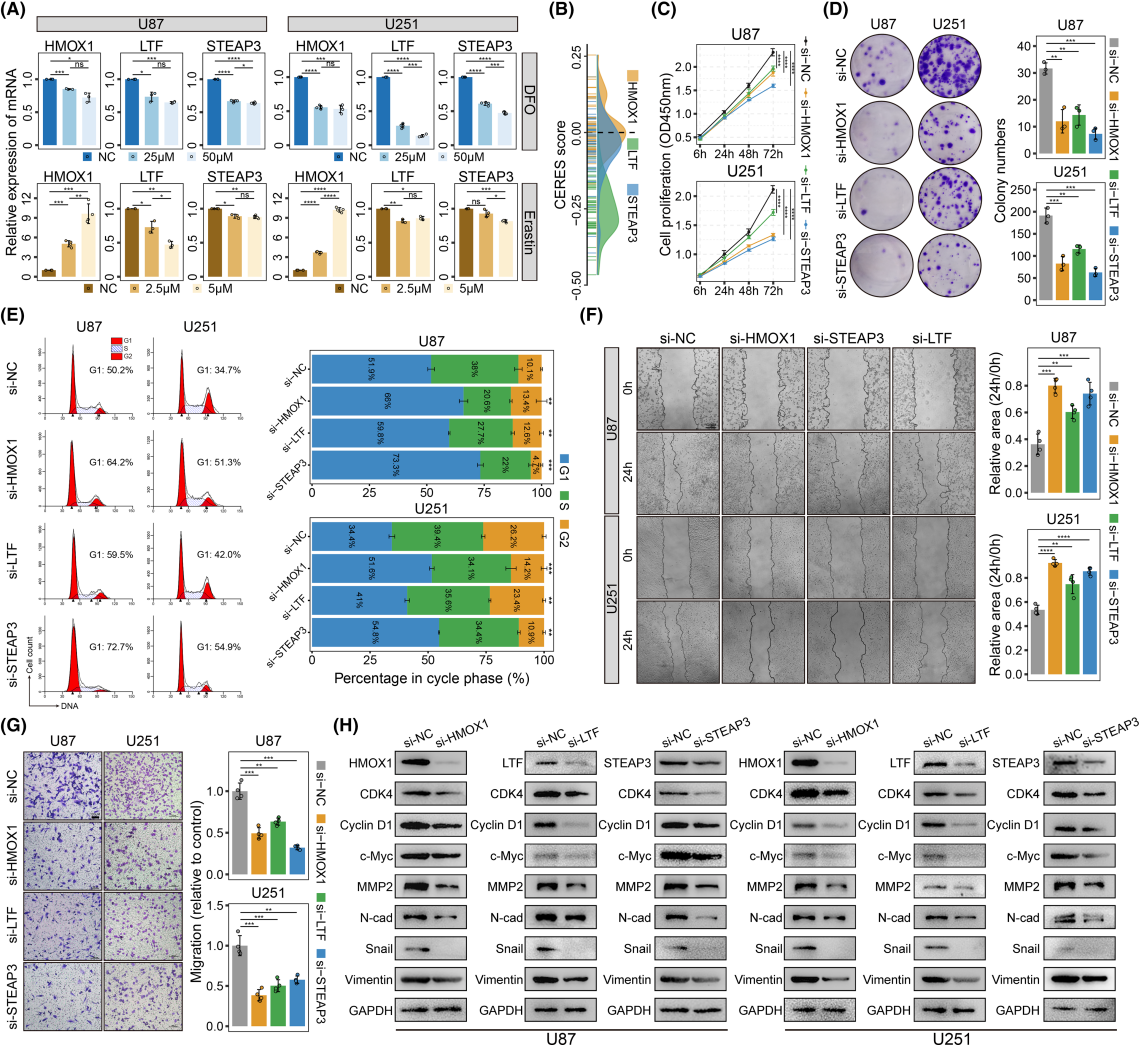
In vitro experiments validated the cellular biological functions of HMOX1, LTF, and STEAP3. (A) mRNA expression levels of the three genes after treatment with different concentrations of erastin and DFO in U87 and U251 cells. Statistical analyses were performed using the Student's *t*‐test. (B) Density plot showing the distribution of CERES scores of the three genes in the DepMap dataset. (C) CCK‐8 assay comparing the OD450 values in U87 and U251 cells transfected with si‐NC, si‐HMOX1, si‐LTF, and si‐STEAP3 after 72 h. *P*‐values were calculated using Student's *t*‐test. (D) Colony formation assay showing changes in the clonogenic formation capacity after knocking down HMOX1, LTF, and STEAP3 in glioma cells. *P*‐values were calculated using the Student's *t*‐test. (E) Representative results showing cell cycle phase quantification in U87 and U251 cells treated with different siRNAs (left panel). Stacked boxplots visualizing the percentages of G1, S, and G2 phases in different groups (right panel). *P*‐values were calculated to compare the percentages in the G2 phase using the Student's *t*‐test. The abilities of U87 and U251 cells for migration (F) and invasion (G) were significantly inhibited after decreasing HMOX1, LTF, and STEAP3 expressions. Scale bar: 100 μm. *P*‐values were calculated using the Student's *t*‐test. (H) Protein expression changes for proliferation‐, migration‐, and invasion‐related molecules after knocking down HMOX1, LTF, and STEAP3 in glioma cells. All data are presented as means ± standard deviation. *p < 0.05, **p < 0.1, ***p < 0.001, ****p < 0.0001.

Since deferoxamine (DFO) and erastin are known to affect intracellular iron concentration and metabolism, we used them as positive controls to determine whether these three genes could regulate iron metabolism in glioma cells. After treatment with different doses of DFO and erastin, the transcriptional activities of HMOX1, LTF, and STEAP3 were reduced in both U87 and U251 glioma cells, indicating that these genes played a role in iron metabolism regulation (Figure 9A).

We then explored whether these three genes affected the proliferation of human glioma cells using DepMap CRISPR‐Cas9 knockout gene effect scores. A total of 59 uncontaminated glioma cell lines with gene effect scores were obtained. CERES scores of 98.305% (58/59), 71.186% (42/59), and 45.763% (27/59) cells were negative in glioma cells after LTF, STEAP3, and HMOX1 knockout, respectively (Figure 9B). GSEA results in both TCGA and CGGA glioma datasets showed that cell proliferation and cell cycle‐related signaling pathways were significantly enriched in samples with HMOX1, LTF, and STEAP3 overexpression (Figure S9A–F).CCK‐8 and colony formation assays also confirmed that proliferation and colony formation of both U87 and U251 cells were considerably hindered after HMOX1, LTF, and STEAP3 knockdown (Figure 9C,D). Flow cytometry showed that knockdown of the three genes induced cell cycle arrest and significantly increased the proportion of cells in G1 phase (Figure 9E). Previous studies have demonstrated that CDK4 and cyclin D1 are both key modulators promoting the G1 transition in glioma cells. Western blotting showed that CDK4 and cyclin D1 protein levels were reduced in U87 and U251 cells following HMOX1, LTF, and STEAP3 knockdown compared to the si‐NC group (Figure 9H). In addition, the expression of c‐Myc, a transcription factor that promotes gliomagenesis, was significantly inhibited by knockdown of the three genes.

Cell migration and invasion‐related gene terms, including epithelial‐mesenchymal transition (EMT), extracellular matrix organization, and cancer invasiveness signature, were also enriched in samples with high expressions of the three genes (Figure S9A–F). Wound closure assays demonstrated that decreased expression of the three genes suppressed U87 and U251 cell migrations (Figure 9F). Fewer glioma cells in the lower wells of knockdown groups compared to the si‐NC group indicated that the loss of function of the three genes impeded malignant invasion of glioma cells (Figure 9G). The expression levels of tumor invasion and EMT‐related molecules, including MMP2, N‐cadherin, Snail, and Vimentin, were significantly decreased in the knockdown glioma groups (Figure 9H). These in vitro findings demonstrated that HMOX1, LTF, and STEAP3 could facilitate the malignant progression of glioma cells.

## 4 DISCUSSION

Recent global efforts to explore the role of iron metabolism in the progression of neoplastic human tumors have revolutionized our understanding and led to the development of several therapeutic agents, including DFO, triapine, and curcumin.^52^, ^53^ However, almost all phase I and II clinical trials using these drugs have failed to demonstrate any improvements in the prognosis of various cancer types, including hepatocellular carcinomas, renal cell carcinomas, and neck squamous cell carcinomas.^54^, ^55^, ^56^ The development of novel therapeutic strategies targeting iron metabolism signaling pathways for gliomas has encountered several obstacles.

Large‐scale high‐throughput analyses have facilitated the discovery of key genomic events in gliomagenesis. Since most of the pioneering studies on iron metabolism in gliomas were based on single genes and limited samples, the present study incorporated transcriptome profiling data from numerous public glioma databases. Gene expression data were obtained using multiple profiling platforms, including microarrays, next‐generation sequencing, and NanoString nCounter, allowing results to be validated in independent cross‐platform and trans‐ethnic glioma cohorts. We performed WGCNA analysis, an algorithm designed to identify module eigengenes associated with specific phenotypes, to discover the overall survival‐related gene module. WGCNA analysis has been widely used in oncological research. Niemira et al. identified CTLA4, MZB1, NIP7, and GNG11 as genes significantly associated with the malignant progression of nonsmall cell lung cancer.^57^ Bai et al. used WGCNA to identify 21 key genes that contributed to tumor stemness in liver cancers.^58^ In the present study, we combined the identified brown module with differentially expressed genes and prognostic molecules; the seven intersecting genes were selected as key IMRGs for gliomas. To strike a balance between the size of the gene signature and the efficacy of clinical translation, less important genes were eliminated using a machine learning method. STEAP3, a membrane protein with a cytoplasmic oxidoreductase structural domain and a C‐terminal heme‐containing transmembrane structural domain, acts as a ferric reductase to reduce Fe^3+^ to Fe^2+^ and regulate cellular iron uptake.^59^ A previous study showed that STEAP3 was overexpressed in colorectal cancer tissues and maintained the intracellular iron storage to promote proliferation.^60^ A recent study by Wang et al. revealed that STEAP3 promotes hepatocellular carcinoma proliferation via the RAC1‐ERK‐STAT3 signaling pathway.^61^ HMOX1 is an isoform of heme oxygenase that functions as a rate‐limiting enzyme, catalyzes heme degradation, and regulates intracellular iron cycling.^62^ HMOX1 has been shown to be associated with nonmuscle invasive bladder cancer recurrence, cisplatin resistance in muscle invasive bladder cancers, and microenvironment remodeling in melanomas.^63^, ^64^, ^65^ As an active iron‐binding protein from the transferrin family, LTF exhibits tumor suppressive effects in various cancer types. It induces G1 phase arrest and inhibits proliferation in breast carcinoma cells.^66^ Ni et al. found that LTF significantly diminishes the metastasis ability of clear cell renal cell carcinomas.^67^ In the present study, however, LTF was overexpressed in glioma samples and was indispensable for the malignant phenotypes in the in silico and in vitro experiments.

We performed a meta‐analysis to estimate the prognostic value of the gene signature in the pan‐glioma cohort, which included both LGG and GBM datasets. The significant heterogeneity indicated variations among studies. We speculated that the heterogeneity may have resulted from differences in overall survival between LGG and GBM patients. Subgroup analyses confirmed that there was no heterogeneity within the groups and that the pooled effect sizes in the two subgroups differed significantly from each other. Our findings demonstrated the robustness and transportability of the gene signature in different glioma cohorts. We also successfully designed and validated a nomogram tool to quantitatively predict the prognosis of glioma patients. Among the clinicopathological and molecular factors, the scoring weight of the risk signature exceeded that of IDH mutation, 1p/19q codeletion, and MGMT methylation, indicating the potential of the IMRG‐based model as a valuable prognostic indicator.

It is well established that the molecular classification of GBMs is based on the transcriptional profiles of glioma‐intrinsic genes.^7^ Mesenchymal GBMs are characterized by a high frequency of NF1 mutations, tumor necrosis, a complex tumor microenvironment, and a poorer prognosis.^7^, ^68^ The transition from other molecular subtypes to the mesenchymal phenotype is strongly associated with malignant progression and is, to some extent, similar to EMT behavior.^69^ Guan et al. demonstrated that gene expression‐based classification can also be used to stratify LGGs, and that LGGs had the same subtypes as GBMs with common molecular features.^70^ Therefore, we predicted the subtype of each sample in the pan‐glioma cohort to explore the intrinsic connection between the IMRG‐based signature and the mesenchymal phenotype. Significant enrichment of the mesenchymal gene term and strong positive correlations between CD44 and the signature genes indicated that the IMRG‐based signature was an effective biomarker for mesenchymal gliomas. However, this finding was based on gene expression data from bulk tumors contaminated by other cell components, including macrophages, neutrophils, and T cells.^68^ Therefore, we included the transcriptome data of patient‐derived and conventional glioma cells from three independent datasets to further validate our findings at the cellular level. A limitation to the application of the three signature genes as novel biomarkers of mesenchymal gliomas was that flow cytometry costaining was not performed in the present study. This method requires live primary glioma cells derived from a considerable number of fresh tumor samples after gene profiling and pre‐stratification into distinct subtypes based on unsupervised clustering. Further flow cytometry experiments are required to validate the coexpression patterns between CD44 and the three signature genes. Single‐cell analysis of data from purified malignant glioma cells showed that HMOX1, LTF, and STEAP3 were predominantly expressed in mesenchymal‐like cell clusters facilitating intratumoral mesenchymal shift over time.

The use of drug sensitivity data from large‐scale tumor cell lines to provide opportunities for therapeutic discovery is promising. Yang et al. identified homoharringtonine as a candidate therapeutic agent for liver cancers using the Library of Integrated Network‐based Cellular Signatures dataset.^71^ Kuenzi et al. developed an interpretable deep learning model to guide drug combinations based on drug sensitivity data from the CTRP and Genomics of Drug Sensitivity in Cancer datasets.^72^ A recent study demonstrated that ridge regression, a regularized linear regression method, outperformed other machine learning models in reflecting clinical drug response.^73^ Therefore, we predicted the sensitivity of glioma patients to TMZ using a ridge regression model based on the GSE91932, CTRP, and PRISM datasets. However, the estimated drug sensitivity data may not fully reflect actual intratumor effects, which is not surprising in view of the highly heterogeneous tumor microenvironment and complex niche. Thus, we used the Ivy‐GAP cohort, which includes details of chemotherapeutic timepoints and corresponding MRI images of GBM patients, to investigate the relationship between the IMRG‐based gene signature and the TMZ response. Based on the Response Assessment in Neuro‐Oncology criteria, we used T1 contrast‐enhanced sequences to show the enhanced tumor region, while T2 FLAIR nonenhancing sequences were used to reflect tumor progression.^74^ Our findings indicated that a high risk level was associated with TMZ resistance, which was consistent with previous results from TMZ sensitivity estimation analyses. The determination of adequate numbers of cysteine‐phosphate‐guanine islands to represent MGMT transcription status has been debated in methylation detection assays.^75^ Furthermore, the use of MGMT methylation status as an indicator for TMZ response is controversial because of the lack of consensus on the optimal methylation testing method.^76^ A retrospective study of 334 GBM patients reported that methylation of the MGMT promoter failed to predict the TMZ response.^77^ Our findings suggest that the gene signature predicted TMZ therapy response more accurately than MGMT methylation status. Since this finding was based on limited clinical GBM samples, further extensive studies are required to validate the predictive value of the gene signature for the benefit of TMZ therapy in large cohorts. In vitro and in vivo experiments demonstrated that the knockdown of these three IMRGs significantly enhanced the sensitivity of gliomas to TMZ treatment.

## 5 CONCLUSIONS

Collectively, the present study provided insights from a comprehensive analysis of IMRGs regarding the mechanisms driving malignant progression in gliomas and identified the three most essential genes among them. The novel gene signature developed using these key genes could predict the prognosis of glioma patients. We further revealed that the gene signature was strongly associated with the mesenchymal phenotype and TMZ resistance. We envisage that the construction of the IMRG‐based signature could facilitate novel targeted therapy approaches for the management of gliomas.

## AUTHOR CONTRIBUTIONS

Jiayue Zhang: Conceptualization, Design, Formal analysis, Methodology, Writing ‐ original draft. Liang Zhao: Conceptualization, Data curation, Methodology, Software. Shurui Xuan: Investigation, Validation, Formal analysis, Writing— original draft. Zhiyuan Liu: Investigation, Validation, Visualization. Zhenkun Weng: Data curation, Resources, Visualization. Yu Wang: Software, Investigation. Kexiang Dai: Data curation, Resources. Aihua Gu: Supervision, Writing—review and editing. Peng Zhao: Supervision, Project administration, Funding acquisition, Writing—review and editing. Jiayue Zhang and Liang Zhao should be considered joint first author.

## CONFLICT OF INTEREST STATEMENT

The authors have declared that no competing interest exists.

## Supporting information


**Figure S1.** Identification of gene modules in the TCGA glioma cohort using WGCNA analysis. (A) Clustering dendrogram of glioma samples using hierarchical clustering based on the average linkage method. The red line represents the cutoff for removing outliner samples. (B) Selection of a soft threshold based on signed R‐squared and mean connectivity statistics. (C) Histogram of connectivity distribution when β = 10 (left panel). Linear fit model, indicating that a scale‐free network was developed when β = 10 (right panel). (D) Clustering dendrogram of gene eigengenes based on module similarity. Modules under the red line (0.3) were merged into a single module. (E) A heatmap visualizing the interaction relationships between the modules.
**Figure S2.** Module preservation analysis in 15 independent glioma datasets. Median rank and Z_summary_ statistics of the gene modules in CGGA (A), REMBRANDT (B), Gravendeel (C), Kamoun (D), Lee (E), Phillips (F), Frejie (G), Gorovets (H), Joo (I), Ducray (J), Weller (K), AVAglio (L), ATE (M), GLARIUS (N), and Yanovich (O) cohorts.
**Figure S3.** The expression levels of mRNA and protein abundances of HMOX1, LTF, and STEAP3 in gliomas. Normalized gene expression of HMOX1 (A), LTF (B), and STEAP3 (C) in human cancer cell lines from the GSE57083 dataset. Glioma cells were highlighted in red font. Overall *p*‐values were calculated using the Kruskal–Wallis test. (D) Comparison of protein expressions of the three genes between normal (*n* = 10) and GBM (*n* = 99) samples using proteomic data from the CPTAC dataset. *P*‐values were calculated using the Wilcoxon rank‐sum test. ***p < 0.001, ****p < 0.0001.
**Figure S4.** Sensitivity analysis of the risk model in glioma cohorts. Leave‐one‐out approaches were used to investigate the influence of individual cohorts on overall HR in pan‐glioma (A), GBM (B), and LGG (C) groups.
**Figure S5.** Construction and evaluation of nomograms for glioma patients. Nomograms were developed using the risk model and clinicopathological features to predict the probability of survival at different time points in the TCGA (A) and CGGA (B) cohorts. Calibration curves for measuring the predictive performance of nomograms in the TCGA (C) and CGGA (D) cohorts. *X*‐axes, nomogram‐predicted survival probability; *y*‐axes, the actual survival probability obtained using Kaplan–Meier analysis. The gray dotted line represents the ideal calibration fit.
**Figure S6.** Relationship of the gene signature to glioma heterogeneity and mesenchymal phenotype. (A) Heatmap depicting the associations between increasing risk scores and clinicopathological characteristics in the CGGA cohort. (B) Pearson correlation between the risk score and tumor purity inferred by using ESTIMATE in the CGGA dataset. GSEA results of proneural‐related gene terms in the TCGA (C) and CGGA (E) datasets and mesenchymal glioma signature in the CGGA (D) cohort. ROC curves measuring the performance of the gene signature in predicting mesenchymal (F) and IGS‐23 (G) subtypes in the CGGA dataset. (H) Pearson correlation between expressions of CD44 and the three signature genes. Scatter plots showing pairwise expression distributions of CD44 with HMOX1, LTF, and STEAP3 in the HGCC (I) and GSE91392 (J) datasets. Red points, mesenchymal glioma cells; blue points are proneural glioma cells.
**Figure S7.** Discovery of cell types in the scRNA‐seq dataset. (A) A dot plot showing the percentage and average expression levels of selected gene markers for each cell type. (B) Copy number profile heatmap of oligodendrocytes and putative malignant glioma cells from the inferCNV analysis. Rows are individual cells, and columns are genes ordered by chromosomal locations.
**Figure S8.** Association of risk scores with the mutation landscape and CNAs in the TCGA cohort. Oncoplot shows genes with mutation rates >5% in both high‐risk (A) and low‐risk (B) groups. The bottom panel represents different variant classifications. (C) A scatter plot of Pearson’s correlation and significance between risk scores and CNA numbers.
**Figure S9.** Signaling pathway enrichment of HMOX1, LTF, and STEAP3 in gliomas. GSEA results show multiple oncogenesis‐related gene terms enriched in samples with high expressions of the three genes in the TCGA (A–C) and CGGA (D–F) cohorts.

**Table S1.** Detailed information of included glioma cohorts
**Table S2.** Clinicopathological characteristics of included glioma cohorts
**Table S3.** List of IMRGs
**Table S4.** Differential expression of IMRGs between glioma and normal samples in the TCGA dataset
**Table S5.** The importance index of seven prognostic IMRGs derived from random survival forest model
**Table S6.** Predicted molecular subtypes of TCGA and CGGA glioma samples
**Table S7.** Univariate and multivariate analyses of clinicopathological characteristics and signature with overall survival in glioma cohorts
**Table S8.** Comparison of the clinicopathological characteristics between high‐ and low‐risk groups in TCGA and CGGA cohorts
**Table S9.** Significantly‐mutated genes across different risk groups in TCGA cohort
**Table S10.** The alteration peaks of CNAs in gliomas with different risk levels
**Table S11.** Estimated AUC values of TMZ in TCGA GBM dataset using external drug response data

**Data S1:**Supporting information

## Data Availability

The data that support the findings of this study are available from the corresponding author upon reasonable request.
