# Supplementary material for: Global analysis of iron metabolism‐related genes identifies potential mechanisms of gliomagenesis and reveals novel targets
Source: CNS Neurosci Ther. 2023 Aug 7;30(2):e14386. doi: 10.1111/cns.14386 (PMC10848104; doi:10.1111/cns.14386)
Supplement: Supplementary file 1 — Figure S1. Identification of gene modules in the TCGA glioma cohort using WGCNA analysis. (A) Clustering dendrogram of glioma samples using hierarchical clustering based on the average linkage method. The red line represents the cutoff for removing outliner samples. (B) Selection of a soft threshold based on signed R‐squared and mean connectivity statistics. (C) Histogram of connectivity distribution when β = 10 (left panel). Linear fit model, indicating that a scale‐free network was developed when β = 10 (right panel). (D) Clustering dendrogram of gene eigengenes based on module similarity. Modules under the red line (0.3) were merged into a single module. (E) A heatmap visualizing the interaction relationships between the modules. Figure S2. Module preservation analysis in 15 independent glioma datasets. Median rank and Zsummary statistics of the gene modules in CGGA (A), REMBRANDT (B), Gravendeel (C), Kamoun (D), Lee (E), Phillips (F), Frejie (G), Gorovets (H), Joo (I), Ducray (J), Weller (K), AVAglio (L), ATE (M), GLARIUS (N), and Yanovich (O) cohorts. Figure S3. The expression levels of mRNA and protein abundances of HMOX1, LTF, and STEAP3 in gliomas. Normalized gene expression of HMOX1 (A), LTF (B), and STEAP3 (C) in human cancer cell lines from the GSE57083 dataset. Glioma cells were highlighted in red font. Overall p‐values were calculated using the Kruskal–Wallis test. (D) Comparison of protein expressions of the three genes between normal (n = 10) and GBM (n = 99) samples using proteomic data from the CPTAC dataset. P‐values were calculated using the Wilcoxon rank‐sum test. ***p < 0.001, ****p < 0.0001. Figure S4. Sensitivity analysis of the risk model in glioma cohorts. Leave‐one‐out approaches were used to investigate the influence of individual cohorts on overall HR in pan‐glioma (A), GBM (B), and LGG (C) groups. Figure S5. Construction and evaluation of nomograms for glioma patients. Nomograms were developed using the risk model and clinicopathological [file CNS-30-e14386-s002.docx]

**Global analysis of iron metabolism-related genes identifies potential mechanisms of gliomagenesis and reveals novel targets**

*Jiayue Zhang, et al.*





**Figure S1. Identification of gene modules in the TCGA glioma cohort using WGCNA analysis. (A)** Clustering dendrogram of glioma samples using hierarchical clustering based on the average linkage method. The red line represents the cutoff for removing outliner samples. **(B)** Selection of soft threshold based on signed R-squared and mean connectivity statistics. **(C)** Histogram of connectivity distribution when β = 10 (left panel). Linear fit model indicating that a scale-free network was developed when β = 10 (right panel). **(D)** Clustering dendrogram of gene eigengenes based on module similarity. Modules under the red line (0.3) were merged into a single module. **(E)** Heatmap visualizing the interaction relationship of the modules.


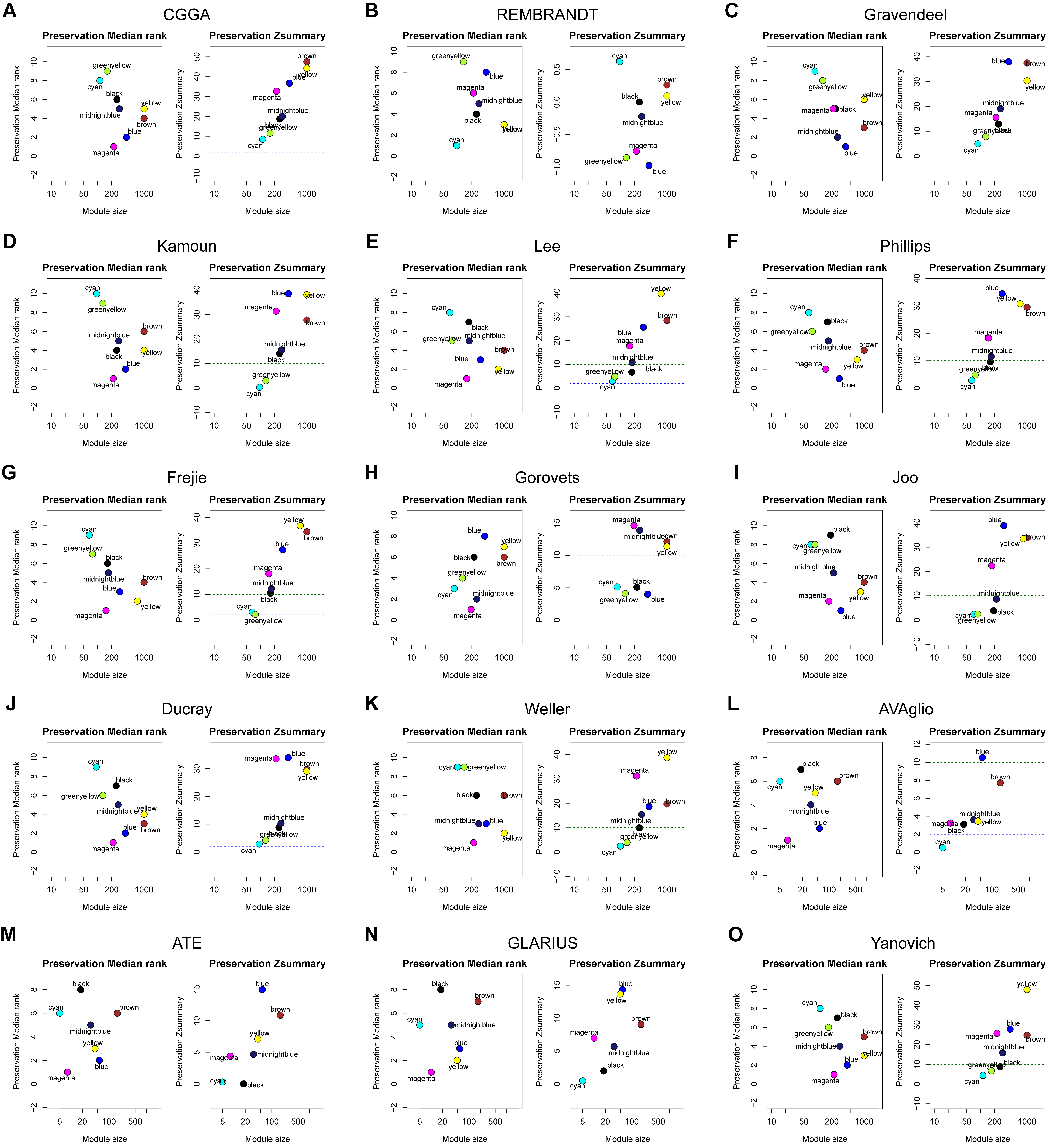


**Figure S2. Module preservation analysis in 15 independent glioma datasets.** Median rank and Z_summary_ statistics of the gene modules in CGGA **(A)**, REMBRANDT **(B)**, Gravendeel **(C)**, Kamoun **(D)**, Lee **(E)**, Phillips **(F)**, Frejie **(G)**, Gorovets **(H)**, Joo **(I)**, Ducray **(J)**, Weller **(K)**, AVAglio **(L)**, ATE **(M)**, GLARIUS **(N)**, and Yanovich **(O)** cohorts.


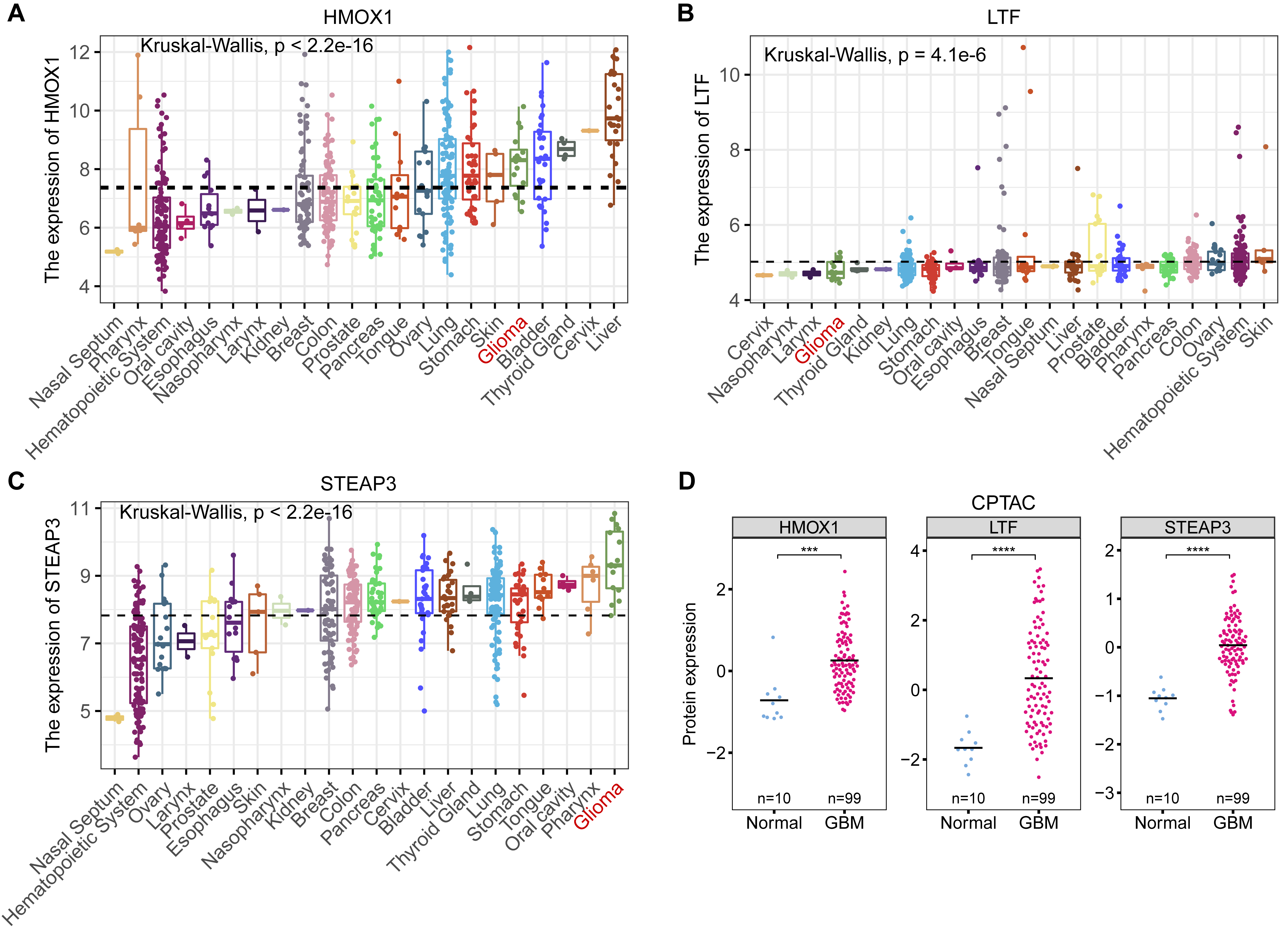


**Figure S3. The expression levels of mRNA and protein abundances of HMOX1, LTF, and STEAP3 in gliomas.** Normalized gene expression of HMOX1 **(A)**, LTF **(B)**, and STEAP3 **(C)** in human cancer cell lines from the GSE57083 dataset. Glioma cells were highlighted in red font. Overall p-values were calculated using Kruskal-Wallis test. **(D)** Comparison of protein expressions of the three genes between normal (n = 10) and GBM (n = 99) samples using proteomic data from the CPTAC dataset. P-values were calculated using the Wilcoxon rank-sum test. *** p < 0.001, **** p < 0.0001.


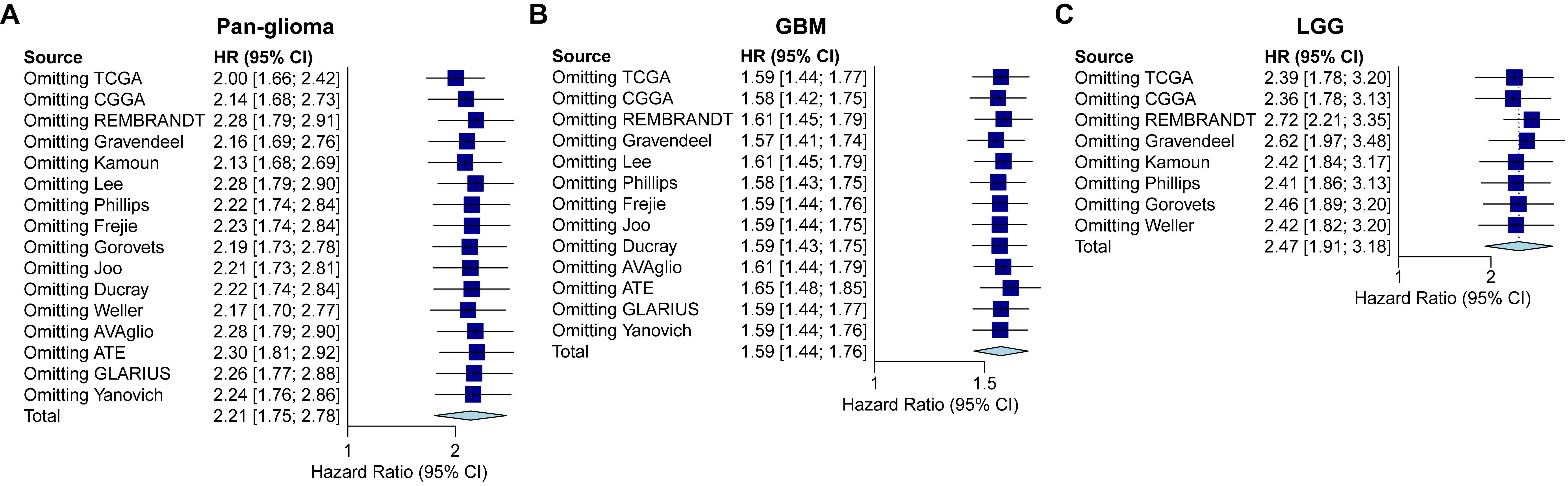


**Figure S4. Sensitivity analysis of the risk model in glioma cohorts.** Leave-one-out approaches were used to investigate the influence of individual cohorts on overall HR in pan-glioma **(A)**, GBM **(B)**, and LGG **(C)** groups.


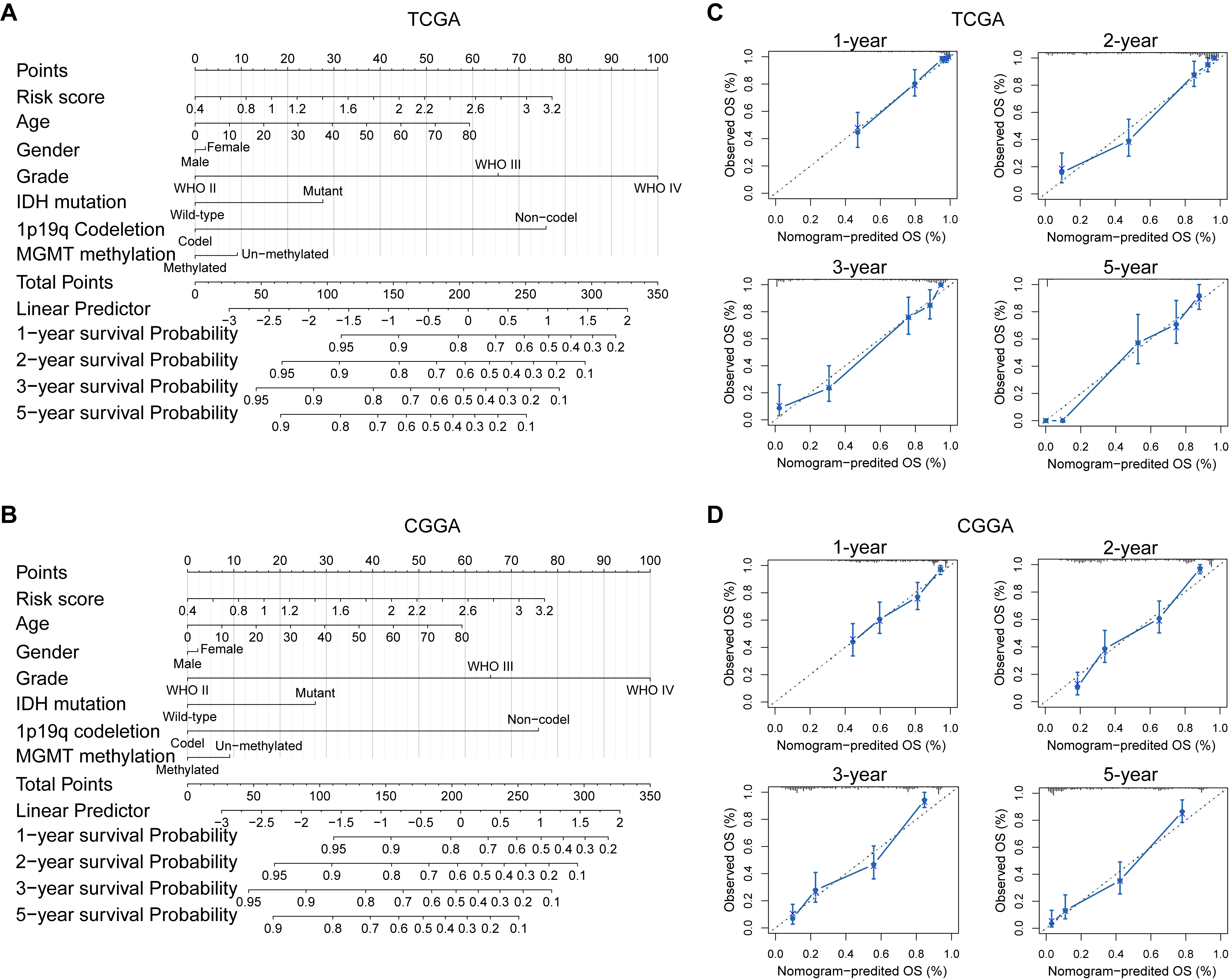


**Figure S5. Construction and evaluation of nomograms for glioma patients.** Nomograms developed using the risk model and clinicopathological features to predict the probability of survival at different time points in TCGA **(A)** and CGGA **(B)** cohorts. Calibration curves for measuring the predictive performance of nomograms in TCGA **(C)** and CGGA **(D)** cohorts. *X*-axes, nomogram-predicted survival probability; *y*-axes, the actual survival probability obtained using Kaplan-Meier analysis. The gray dotted line represents the ideal calibration fit.


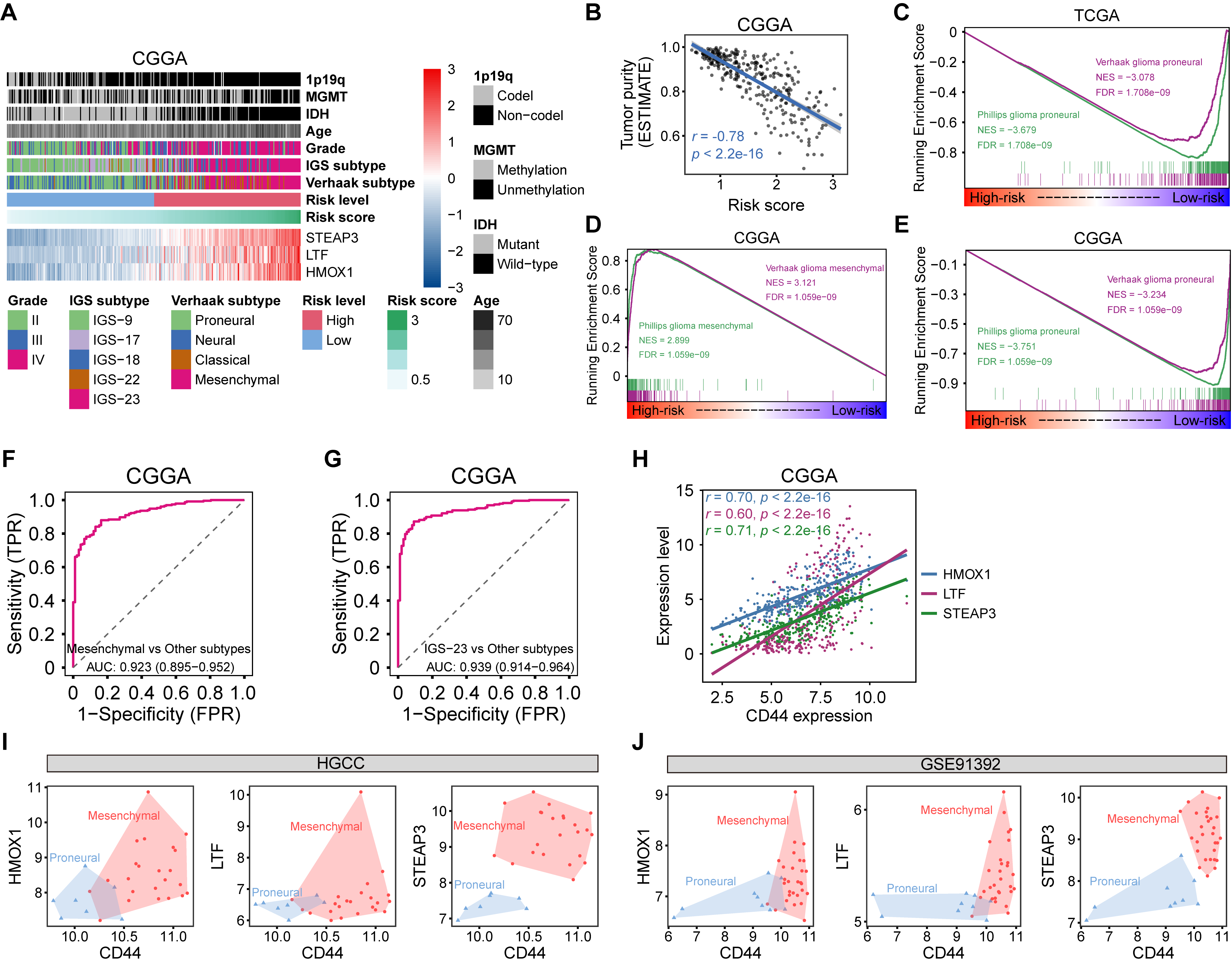


**Figure S6. Relationship of the gene signature to glioma heterogeneity and mesenchymal phenotype. (A)** Heatmap depicting the associations between increasing risk score and clinicopathological characteristics in the CGGA cohort. **(B)** Pearson correlation between the risk score and tumor purity inferred by using ESTIMATE in the CGGA dataset. GSEA results of proneural-related gene terms in TCGA **(C)** and CGGA **(E)** datasets, and mesenchymal glioma signature in the CGGA **(D)** cohort. ROC curves measuring performance of the gene signature in predicting mesenchymal **(F)** and IGS-23 **(G)** subtypes in the CGGA dataset. **(H)** Pearson correlation between expressions of CD44 and the three signature genes. Scatter plots showing pairwise expression distribution of CD44 with HMOX1, LTF, and STEAP3 in HGCC **(I)** and GSE91392 **(J)** datasets. Red points, mesenchymal glioma cells; blue points, proneural glioma cells.


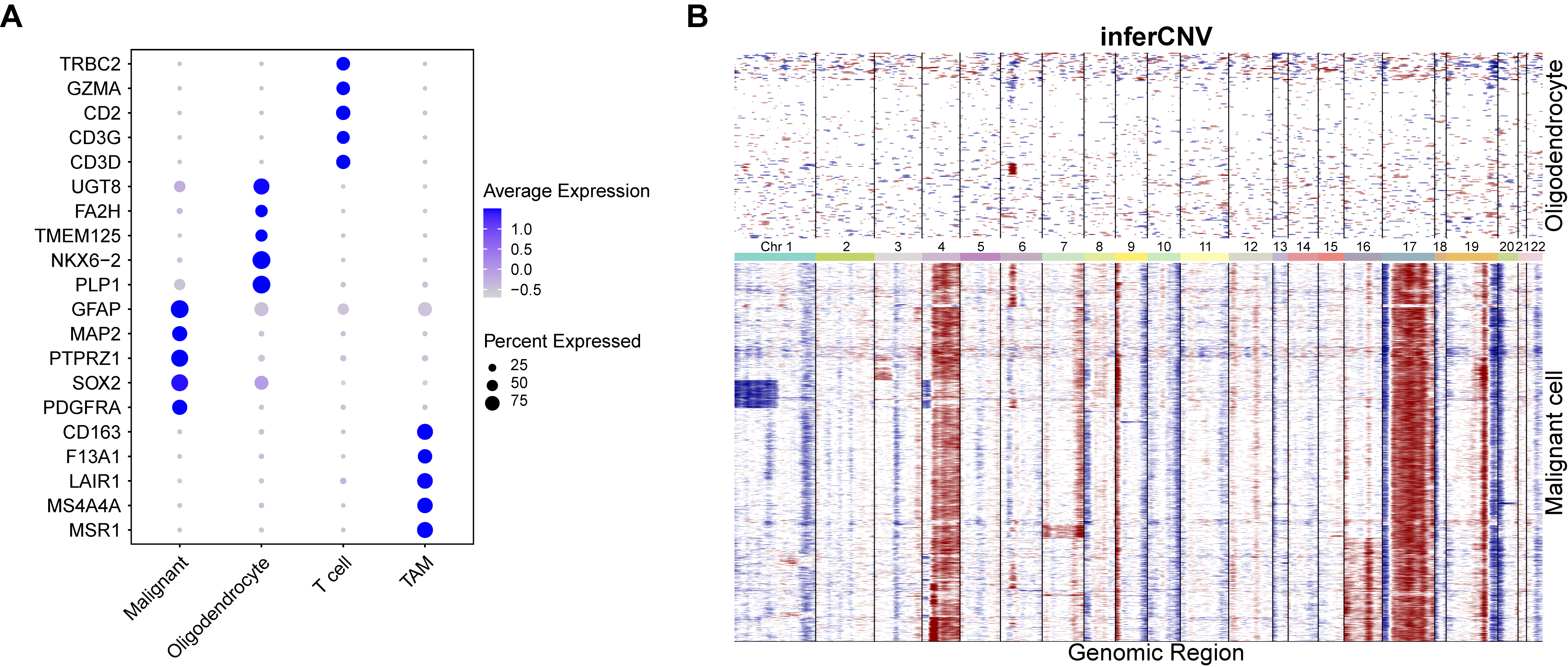


**Figure S7. Discovery of cell types in the scRNA-seq dataset. (A)** Dot plot showing the percentage and average expression levels of selected gene markers for each cell type. **(B)** Copy number profiles heatmap of oligodendrocytes and putative malignant glioma cells from the inferCNV analysis. Rows are individual cells and columns are genes ordered by chromosomal locations.


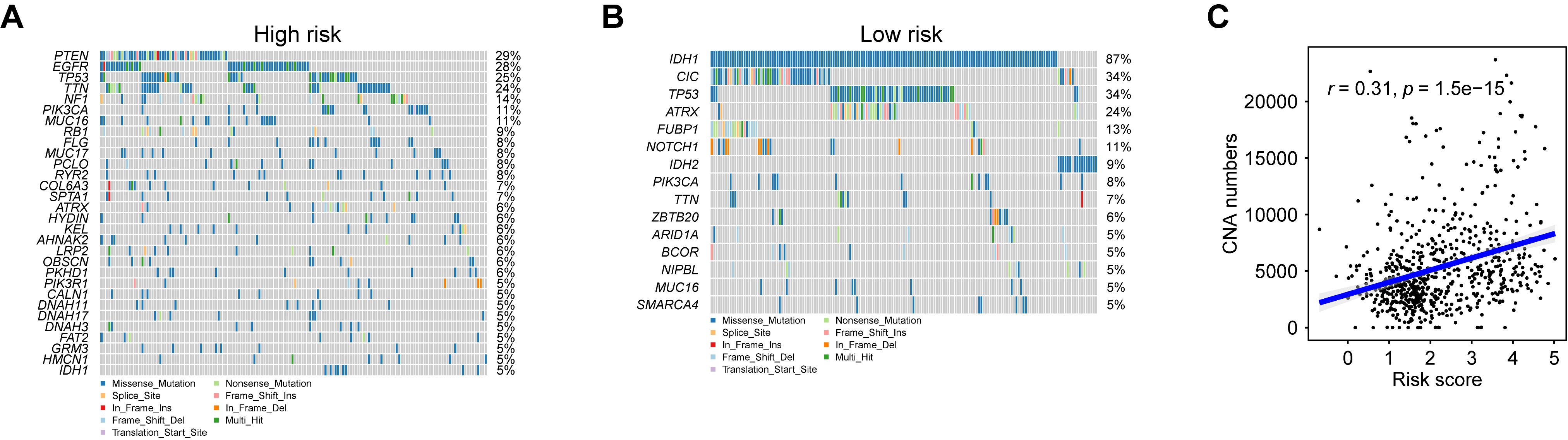


**Figure S8. Association of risk scores with the mutation landscape and CNAs in the TCGA cohort.** Oncoplot showing genes with mutation rate > 5% in both high- **(A)** and low-risk **(B)** groups. The bottom panel represents different variant classifications. **(C)** Scatter plot of Pearson's correlation and significance between risk scores and CNA numbers.


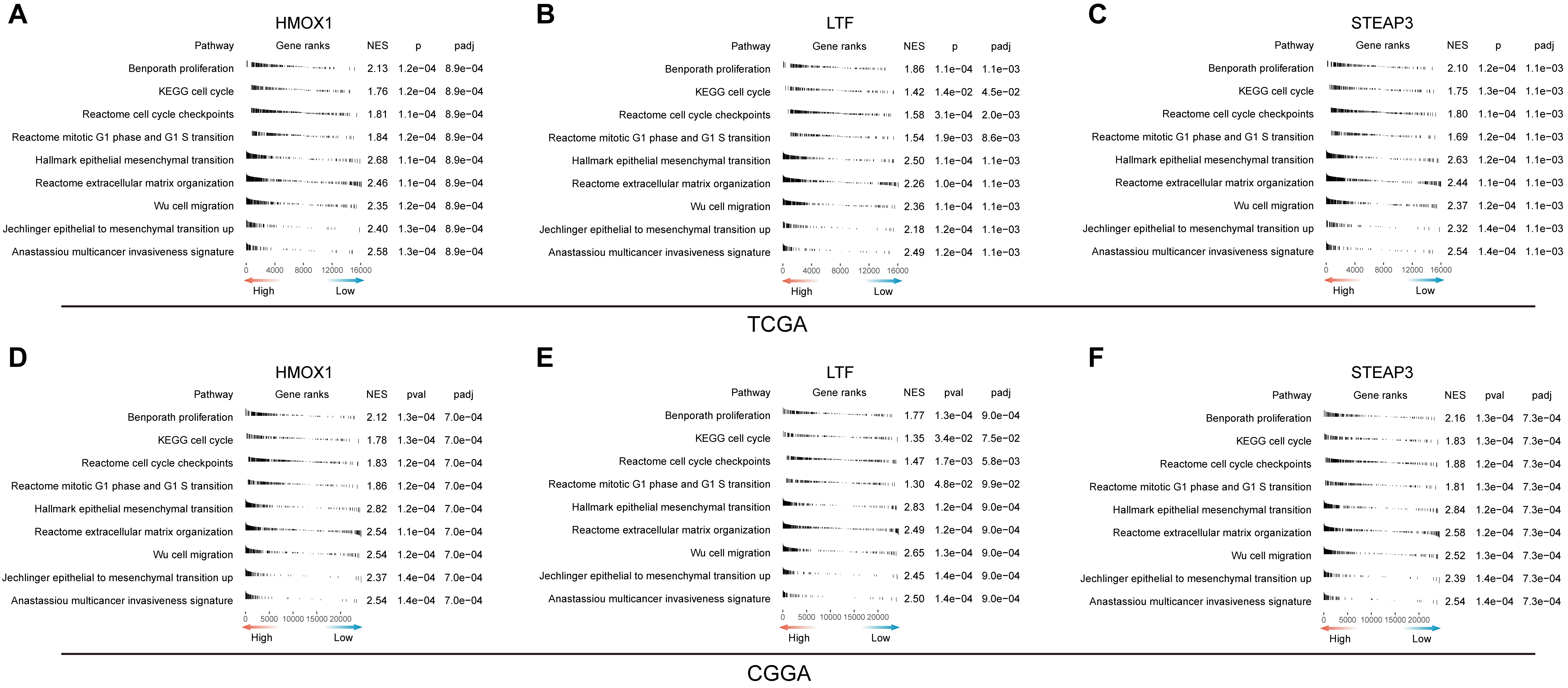


**Figure S9. Signaling pathway enrichment of HMOX1, LTF, and STEAP3 in gliomas.** GSEA results showing multiple oncogenesis-related gene terms enriched in samples with high expressions of the three genes in TCGA **(A-C)** and CGGA **(D-F)** cohorts.
