# Supplementary material for: Global analysis of iron metabolism‐related genes identifies potential mechanisms of gliomagenesis and reveals novel targets
Source: CNS Neurosci Ther. 2023 Aug 7;30(2):e14386. doi: 10.1111/cns.14386 (PMC10848104; doi:10.1111/cns.14386)
Supplement: Supplementary file 3 — Data S1:Supporting information [file CNS-30-e14386-s003.zip › Supplementary methods_revised.docx]

**Supplementary methods**

**Global analysis of iron metabolism-related genes identifies potential mechanisms of gliomagenesis and reveals novel targets**

*Jiayue Zhang, et al.*

**Material and methods**

**Patient samples collection**

Twenty archival paraﬃn-embedded tissues, including 15 glioma samples (five cases for each grade) and five normal brain samples from cerebral trauma, were collected from the Department of Neurosurgery, the First Aﬃliated Hospital of Nanjing Medical University. The diagnosis and tumor grade of each sample was confirmed by an experienced pathologist. All donors provided informed consent. The study was approved by the ethics committee of the First Aﬃliated Hospital of Nanjing Medical University (ethics number: 2020-SRFA-172) and conducted according to the principles of the Declaration of Helsinki.

**Immunohistochemistry (IHC) staining**

The paraffin-embedded tissues were sliced, deparaffinized, and then received antigen retrieval. The slices were incubated with primary antibodies against HMOX1 (#10701-1-AP, Proteintech, Wuhai, China, 1:100 dilution), LTF (#ab15811, Abcam, Cambridge, UK, 1:100 dilution), and STEAP3 (#17186-1-AP, Proteintech, Wuhai, China, 1:200 dilution) respectively overnight at 4 ℃. The sections were further incubated with a specific second antibody (goat anti-rabbit IgG). Finally, the slices were performed diaminobenzidine reaction and hematoxylin staining. Images were captured using the Panoramic digital slide scanner (3DHISTECH, Budapest, Hungary) according to the manufacturer’s instructions. The positive staining rate of the selected region in each slide was quantified using open-source pathological image analysis software Qupath (v0.2.3, https://qupath.github.io/).

**Cell culture and treatment**

Human glioma cell lines U87 and U251 were purchased from the National Collection of Authenticated Cell Cultures (Shanghai, China) and cultured in Dulbecco’s modiﬁed Eagle’s medium (DMEM; Thermo Fisher, USA) supplemented with 10% fetal bovine serum (FBS; Gibco, USA). All cell lines were maintained with 5% CO_2_ at 37 ℃ in a humidiﬁed atmosphere. Deferoxamine (#D9533, Sigma-Aldrich, USA) and erastin (#S7242, Selleck Chemicals, USA) were diluted in different concentrations and incubated with both U87 and U251 cells, respectively, for 48h. After that, cells were harvested for RNA isolation.

**Quantitative real-time polymerase chain reaction (qRT-PCR)**

Total cellular RNA was extracted from cells using TRIzol reagent (Invitrogen, USA), and cDNA was synthesized using a reverse transcription-PCR Kit (Roche, Switzerland). The reverse transcribed cDNA was further amplification with LightCycler 480 System (Roche, Switzerland). The primer sequences of the target genes were listed as follows: HMOX1 primers, forward: 5′-AAGACTGCGTTCCTGCTCAAC-3′, reverse: 5′-AAAGCCCTACAGCAACTGTCG-3′; LTF primers, forward: 5′-ATGGTGGTTTCATATACGAGGCA-3′, reverse: 5′-CTTTCGGTCCCGTAGACTTCC-3′; STEAP3 primers, forward: 5′-CTCCCCGGAGGTCATCTTTG-3′, reverse: 5′-TCTTGCTCTGTAGGGTTGCTC-3′; GAPDH primers, forward: 5′-GGCACAGTCAAGGCTGAGAATG-3′, reverse: 5′-ATGGTGGTGAAGACGCCAGTA-3′. The difference of relative quantitative value for each gene in different groups was analyzed using the 2^−ΔΔct^ method.

**siRNA transfection**

Glioma cells were seeded overnight and then transfected with siRNAs using Lipofectamine 3000 reagent (Thermo Fisher, USA) when the cell density reached 30%-50%. The siRNAs targeting HMOX1, LTF, and STEAP3 were designed and synthesized by RiboBio (Guangzhou, China). The target sequences of the siRNAs were listed as follows: HMOX1: 5′-CCAGCAACAAAGUGCAAGAdTdT-3′, LTF: 5′-GGGACGAGTATGAGTTACT-3′, STEAP3: 5′-GCUUCUAUGCCUACAACUU -3′.

**Immunoﬂuorescence (IF) staining**

U87 and U251 glioma cells were fixed with 4% paraformaldehyde and blocked with a solution of 5% BSA and 0.01% Triton-X 100. Then, the cells were incubated with primary antibodies overnight, washed with PBS, and further incubated with appropriate fluorescence-conjugated secondary antibodies. DAPI (Invitrogen, USA) was used to counterstain the nuclei for 5 min. Images were taken using a ﬂuorescence confocal microscope (CarlZeiss, Germany). Images were processed with the software ZEN 3.1 (blue edition). The antibodies used in this assay were listed as follows: HMOX1 (#10701-1-AP, Proteintech, Wuhai, China, 1:200 dilution), LTF (#ab15811, Abcam, Cambridge, UK, 1:100 dilution), STEAP3 (#17186-1-AP, Proteintech, Wuhai, China, 1:200 dilution), CD44 (#3570, Cell Signaling Technology, USA, 1:200 dilution).

**Cell viability assay**

Glioma cells were seeded in 96-well plates at 4*10^3^ per well in 100μl complete culture medium. Then 10μl Cell Counting Kit-8 reagent (CCK-8, Dojindo, Japan) was added into each well after 6h, 24h, 48h, and 72h of culture. After that, cultures were incubated for 2 h at 37 ℃. The absorbance of each well (OD value) was measured at a wavelength of 450 nm.

For the temozolomide (TMZ) sensitivity test, pre-treated glioma cells with targeted knockdown of different genes using siRNAs were added to a 96-well plate, followed by the treatment with TMZ (HY-17364, MedChemExpress, Sweden) at various concentrations (0-1600μM, dissolved in DMSO). After routine culture for 48 hours, the OD values were measured. The half-maximal inhibitory concentration (IC50) value for each group was calculated using a four-parameter logistic nonlinear regression model based on the OD values.

**Colony formation assay**

A total of 500 glioma cells were seeded in each 35mm dish and cultured for 2 weeks in DMEM supplemented with 10% of FBS. The colonies were washed with PBS, fixed with 4% formaldehyde, and stained with 0.1% crystal violet (Beyotime, Shanghai, China).

**Flow cytometry**

Cells were dissociated into single cells with trypsin, resuspended in PBS, and fixed by 70% ice-cold ethanol at 4℃. Then, cells were washed with PBS and stained with propidium iodide (Santa Cruz Biotechnology, USA). Cell cycle progression was evaluated using FACS Calibur flow cytometer (BD Biosciences, USA) and analyzed with ModFitLT software (v3.2).

*Wound closure assay*

Transfected glioma cells were seeded in 6-well plates and cultured to 90% density. Cells were washed with PBS twice and then scratched with a pipette tip. Cells were washed again with PBS to remove dissociated cellular fragments and then cultured in a medium with 1% FBS. Images were photographed using inversion microscopy (Olympus, Japan) at different time points.

**Transwell invasion assay**

Pre-melted matrigel matrix (#356234, Corning, USA) was added onto the upper layer of the transwell membrane (#3422, Corning, USA) and placed in the incubator for 30 min. Cells were resuspended in serum-free medium and then seeded in the upper compartment of the insert at 5*10^3^ per well. A complete medium with 10% FBS was added to the well of the plate. After 24h incubation, cells in the upper layer of the insert were wiped gently. Cells on the lower side of the insert membrane were fixed and stained with 1% crystal violet.

**Western blotting**

In brief, cells were washed with PBS and lysed in RIPA lysis buffer and phenylmethanesulfonyl fluoride (PMSF) on ice for 20 min. The protein concentrations were measured using a BCA assay kit (Thermo Fisher, USA). Equal amount of protein was separated by sodium dodecyl sulfate-polyacrylamide gel electrophoresis (SDS-PAGE) and transferred to a PVDF membrane. The membranes were incubated with primary antibodies overnight at 4℃ and washed with PBS containing 0.1% Tween for 20 min. Membranes were then incubated with horseradish peroxidase-labeled secondary antibodies at room temperature. The blots were visualized by an enhanced chemiluminescence reagent (Millipore, USA). Primary antibodies used in this section were listed as follows: HMOX1 (#10701-1-AP, Proteintech, Wuhai, China, 1:1000 dilution), LTF (#ab15811, Abcam, Cambridge, UK, 1:1000 dilution), STEAP3 (#17186-1-AP, Proteintech, Wuhai, China, 1:1000 dilution), cyclin D1 (#ab134175, Abcam, Cambridge, UK, 1:1000 dilution), c-Myc (#sc-40, Santa Cruz Biotechnology, USA, 1:1000 dilution), MMP2 (#13132, Cell Signaling Technology, USA, 1:1000 dilution), N-cadherin (#13116​, Cell Signaling Technology, USA, 1:1000 dilution), snail (#3879, Cell Signaling Technology, USA, 1:1000 dilution), vimentin (#5741, Cell Signaling Technology, USA, 1:1000 dilution), and GAPDH (#ab8245, Abcam, Cambridge, UK, 1:1000 dilution).

***In vivo* xenograft model**

Five-week-old male BALB/c athymic nude mice were purchased from the Experimental Animal Center of Nanjing Medical University. The animal experiments in this study were carried out according to the protocols approved by the Institutional Animal Care and Use Committee of Nanjing Medical University (IACUC-2304045).

To establish the xenograft tumor model, 5*10^6^ U87 glioma cells were injected into the right axillary subcutaneous region of nude mice (body weight of 20 g). After the formation of solid tumors, nude mice were randomly assigned into five groups (siRNA control, siRNA control+TMZ, si-HMOX1+TMZ, si-STEAP3+TMZ, and si-LTF+TMZ group) and treated with or without TMZ by oral gavage (22 mg/kg, 5 times per week). The tumor volume was measured with caliper every 5 days and calculated using the formula: volume (mm^3^) = (width^2 * length)/2. Simultaneously, the corresponding siRNAs (2' O-methyl+5' cholesterol-modified; 5 nmol; RiboBio, China) targeting HMOX1, STEAP3, and LTF were dissolved in PBS and then injected into the tumor twice a week. For the siRNA control and siRNA control+TMZ groups, only PBS were intratumorally injected. The mice were sacrificed under anesthesia, and the tumors were harvested for further analysis at 25 days.
