# Supplementary figures and images for: Global analysis of iron metabolism‐related genes identifies potential mechanisms of gliomagenesis and reveals novel targets
Source: CNS Neurosci Ther. 2023 Aug 7;30(2):e14386. doi: 10.1111/cns.14386 (PMC10848104; doi:10.1111/cns.14386)

Full unedited blot for Figure 9H

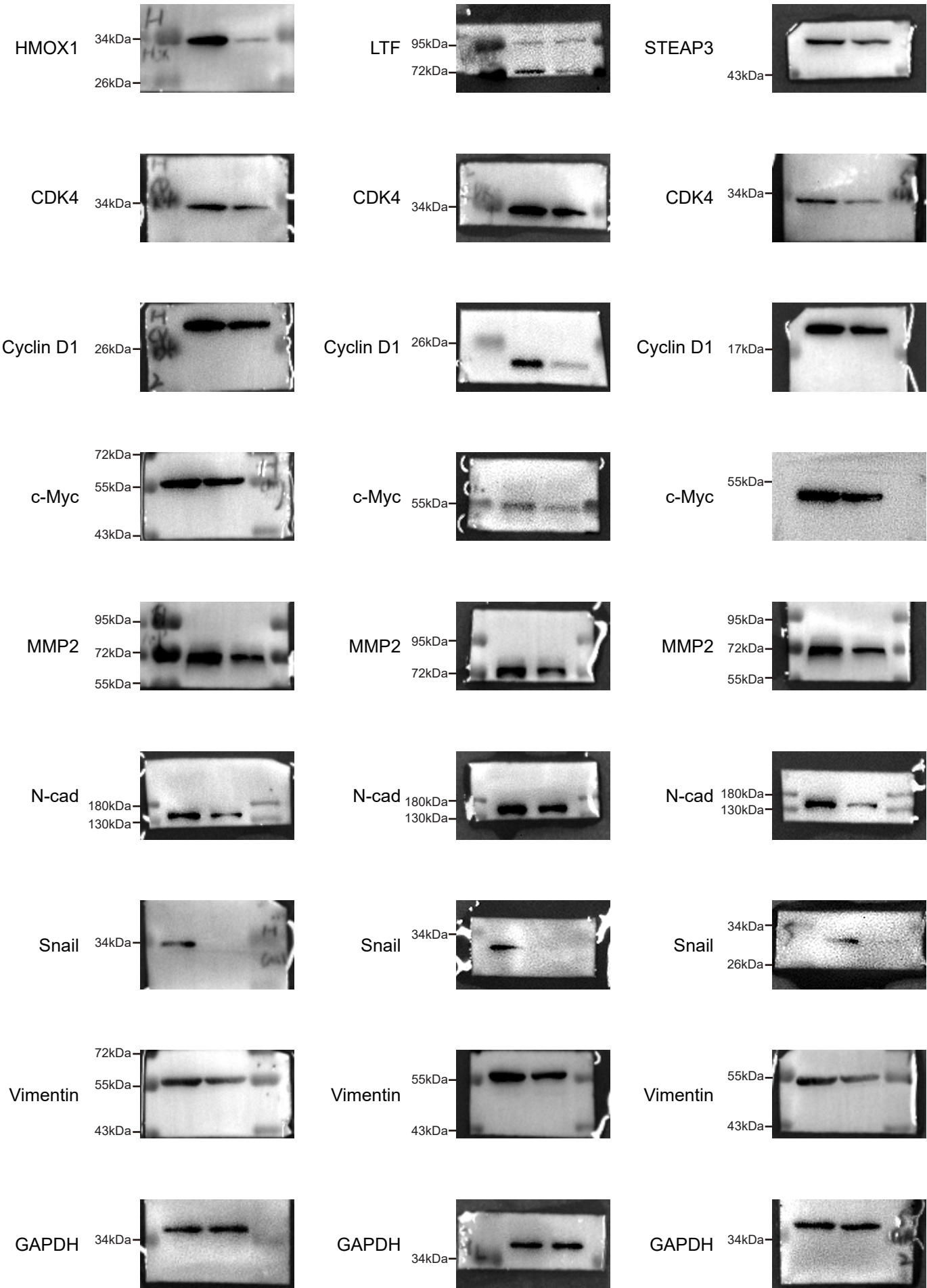

U87

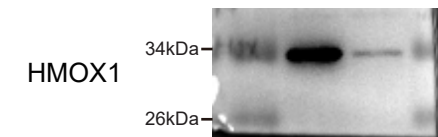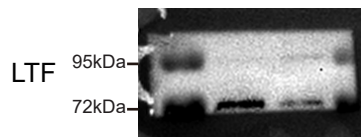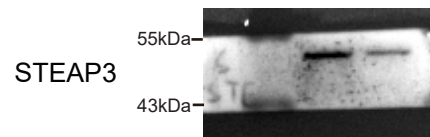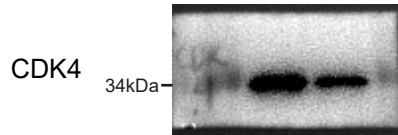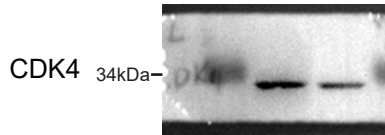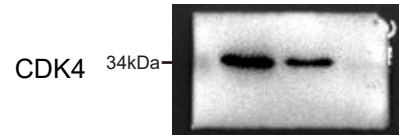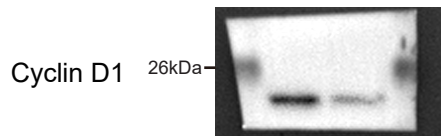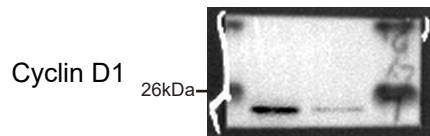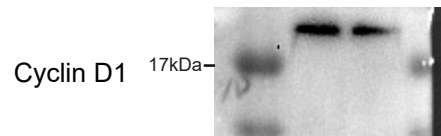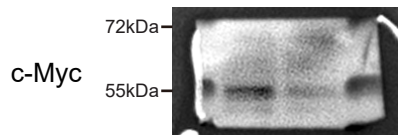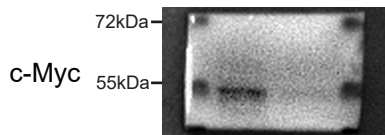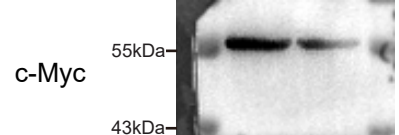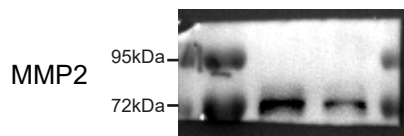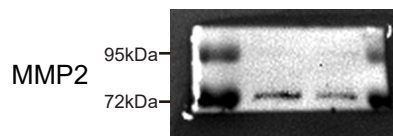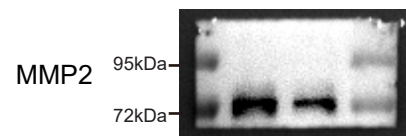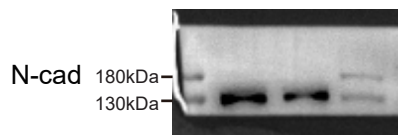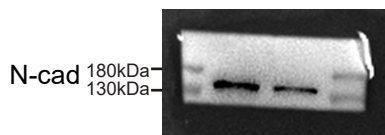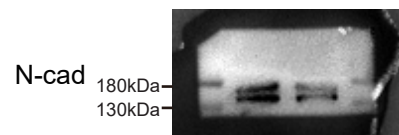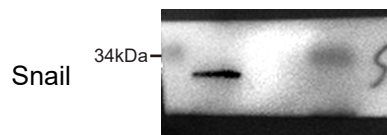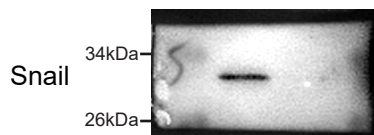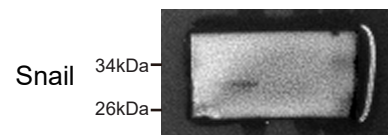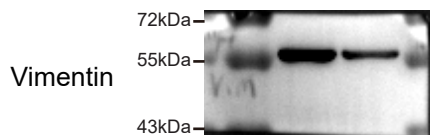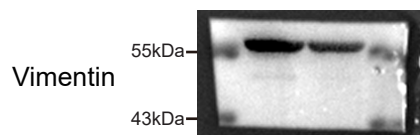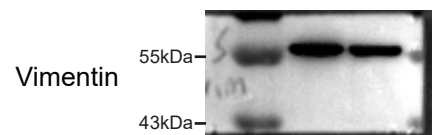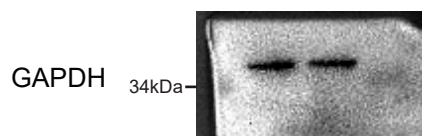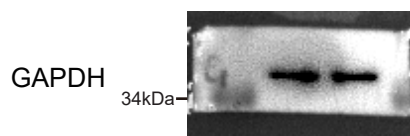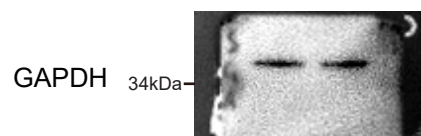

U251

Supplement: Supplementary file 3 — Data S1:Supporting information [file CNS-30-e14386-s003.zip › Supplemental Files_unedited_blots.pdf]
